# *Cardinium* disrupts *Wolbachia*–host dynamics in the domestic mite *Tyrophagus putrescentiae*: evidence from manipulative experiments

**DOI:** 10.1128/msystems.01769-24

**Published:** 2025-04-18

**Authors:** Jan Hubert, Eliza Glowska-Patyniak, Scot E. Dowd, Pavel B. Klimov

**Affiliations:** 1 Department of Microbiology, Nutrition and Dietetics, Faculty of Agrobiology, Food and Natural Resources, Czech University of Life Sciences Prague 48371, Prague, Czechia; 2 Department of Animal Morphology, Faculty of Biology, Adam Mickiewicz University in Poznan, Poznan, Poland; 3 MR DNA (Molecular Research LP), Shallowater, Texas, USA; 4 Purdue University 311308 , West Lafayette, Indiana, USA; Universita degli Studi di Bari Aldo Moro, Bari, Italy

**Keywords:** mite, *Cardinium*, *Wolbachia*, genome, gene expression, interaction

## Abstract

We investigated the tripartite interactions between two intracellular bacterial symbionts, *Cardinium* and *Wolbachia* in *Tyrophagus putrescentiae*. Cultures of *Tyrophagus putrescentiae* are typically single-infected by one intracellular symbiont. However, co-infection can be experimentally induced by mixing single-infected cultures, resulting in 10% of mite individuals being double-infected (*Cardinium + Wolbachia*) and a corresponding reduction in host fitness. Here, we assembled the genomes of *Cardinium* and *Wolbachia* and analyzed their gene expression in parental single-infected and mixed mite cultures using population-level samples (ranging from 7,500 to 10,000 mites). *Wolbachia* interacts more extensively with its mite host than *Cardinium* in single-infected cultures. However, in mixed cultures, (i) *Wolbachia* exhibited reduced regulation of the host compared with *Cardinium*; (ii) the gene expression profile of *Cardinium* shifted, increasing its interactions with the host, whereas the gene expression profile of *Wolbachia* remained unchanged; and (iii) *Wolbachia* genes exhibited a loss of interactions with mite gene expression, as indicated by reduced correlations (for example with host MAPK, endocytosis, and calcium signaling pathways). The experiments show that at the mite population level, symbiont infection disrupts gene expression interaction between the two symbionts and their host in different ways. *Wolbachia* was more influenced by *Cardinium* gene expression than vice versa. *Cardinium* can inhibit the growth of *Wolbachia* by disrupting its interaction with the host, leading to a loss of *Wolbachia’s* influence on mite immune and regulatory pathways. The reasons for responses are due to co-infection or the reduced frequency of *Wolbachia* single-infected individuals due to the analyses of population-level samples.

**IMPORTANCE:**

We found that *Cardinium* disrupts the interaction between *Wolbachia* and mite host. In *Wolbachia* single-infected cultures, strong correlations exist between symbiont and host gene expressions. Interestingly, although *Cardinium* can also interact with the host, this interaction appears weaker compared with *Wolbachia* in single-infected cultures. These results suggest that both symbionts affect mite host gene expression, particularly in immune and regulatory pathways. In mixed samples, *Cardinium* appears to outcompete *Wolbachia* by disrupting its host interaction. It indicates competition between these two intracellular symbionts in mite populations. *Wolbachia* belongs to a mite-specific supergroup Q, distinct from the more commonly studied *Wolbachia* supergroups. As these mite-specific bacteria exhibit pathogen-blocking effects, our findings may have relevance for other systems, such as ticks and tick-borne diseases. The study sheds light on intracellular symbiont interaction within a novel mite-symbiont model.

## INTRODUCTION

The endosymbiotic bacteria *Cardinium* (Cytophagales) and *Wolbachia* (Alphaproteobacteria) are reproductive parasites that affect host reproduction by inducing cytoplasmic incompatibility (CI), feminization, male killing, and parthenogenesis (1–8). Both *Cardinium* and *Wolbachia* are widespread, infecting an estimated 13% and 52% of the terrestrial arthropod species, respectively (9). The genus *Cardinium* is a bacterial lineage of the Fibrobacterota, Chlorobiota, and Bacteroidota (FCB) group belonging to the order Cytophagales that adopted an endosymbiotic lifestyle inside the cytoplasm of eukaryotic multicellular animals (10, 11). *Wolbachia* is another lineage of maternally transmitted intracellular symbionts belonging to phylum Alphaproteobacteria, order Rickettsiales (12).

Some *Wolbachia* strains have evolved to function as beneficial nutritional symbionts, providing biotin (vitamin B_7_) and thereby enhancing host fitness (13). *Cardinium* genomes lack all major biosynthetic pathways except those for peptidoglycan biosynthesis, glycolysis, lipoic acid, and biotin, thus potentially providing the latter two to the host (14, 15). There is evidence that *Wolbachia* influences its hosts via pathogen blocking, which limits the ability of many pathogenic viruses, bacteria, and nematodes to grow in the host (16, 17). Due to a pronounced pathogen-blocking effect, it renders them suitable candidates for the control of mosquito- or vectors of tick-borne diseases (18, 19).

Arthropod hosts may host a single bacterial symbiont species or multiple bacterial species (20–26). Although single infections by *Wolbachia* or *Cardinium* seem to be more typical in mites (27), double infections (*Cardinium* plus *Wolbachia*) have been reported in several plant-feeding mite species in the genera *Bryobia* (28), *Tetranychus* (22, 28–31), and *Panonychus ulmi* (32). To our knowledge, the interaction between *Cardinium* and *Wolbachia* in multi-infected hosts is not well understood, with only a few studies showing the functional effects of their coexistence: (i) altering CI, for example, in *Tetranychus piercei* (22) and *Tetranychus truncatus* (31); (ii) the lack of competition between *Cardinium* and *Wolbachia* in *Pezothrips kellyanus* (33); (iii) promoting fat and free amino acid synthesis in double-infected spider *Hylyphantes graminicola* (34) and interaction in methionine and fatty acid biosynthesis and biotin transport in double-infected nematodes (23); (iv) differences in up-/down-regulation of some genes in *Sogatella furcifera* among single- and double-infected individuals (35); and (v) differently induced pattern recognition receptors and serine protease in silkworms (36). The up-/down-regulation of receptors in host immune pathways (35, 36) indicates that these pathways represent possible targets for the competition between intracellular symbionts. However, there is still a gap in studies incorporating gene expression data from both symbionts and their host. Symbiont co-infections require further investigation.

*Tyrophagus putrescentiae* is a worldwide pest, damaging stored products (37, 38) and producing allergens (39, 40). Until now, four single *Cardinium* (cTPut)*,* two single *Wolbachia* (wTPut)-infected cultures, and four asymbiotic (no cTPut neither wTPut) cultures of *T. putrescentiae* are known, based on barcode sequencing of microbiome and genome analyses (41–46). No natural double-infected (wTPut + cTPut) cultures are known. cTPut and wTPut are maternally transmitted via infected eggs (41, 46); in contrast, potential gut symbionts (*Bartonella*-like, *Blattabacterium*-like, *Solitalea*-like*,* and *Sodalis*-like) are transmitted horizontally through feces (41, 47).

In laboratory settings, mixing single-infected (cTPut and wTPut) mite cultures results in cultures where 100% of mites are infected with cTPut (*N* = 60); however, after 21 days, 25% of these mites are co-infected with both cTPut and wTPut based on single mite PCR with specific primers (46). In another experiment, after 42 days, mixed cultures contained 50% cTPut-infected individuals (*N* = 360), 30% of wTPut-infected individuals, and 10% of double-infected individuals (48). The levels of infection in parental cultures were following cTPut 50% of individuals and wTPut 30% (48). In population-level samples, cTPut reduced wTPut 3-fold based on barcode sequencing and 10-fold based on qPCR on mite microbiome after 21 days of experiment (46). The life cycle of *T. putrescentiae* from egg to adult takes 9.4 days at 25°C but 7.2 days at the optimal temperature of 30°C (49). Population density, used as a fitness indicator (50), was 30% lower in mixed cultures compared with single-infected parent populations after 21 days of the experiment (46). CI can be caused by *Wolbachia* and *Cardinium,* whereas bidirectional CI caused by both symbionts should be present in *T. putrescentiae* (48). Bidirectional CI was described for more *Wolbachia* linkages infected with the same host (51). The data indicate that cTPut is present in both males and females (48). The proportion of wTPut-infected individuals did not statistically differ from the proportion of females, indicating that wTPut infected only *T. putrescentiae* females (48, 52). The condition for CI existence is the presence of a symbiont in the males, which modifies sperm, leading to embryonic mortality in crosses with *Wolbachia*-free females (53). It provides a hypothesis that cTPut has responsibility for CI and remains the most possible explanation for fitness decrease (46, 48).

The purpose of the study was to elucidate the differences in the effects of *Cardinium* and *Wolbachia* on a shared mite host, focusing on host gene expression using mite population-level meta-transcriptomic data.

## RESULTS

### Genomic aspects of *Cardinium* and *Wolbachia*

**Fig 1:**
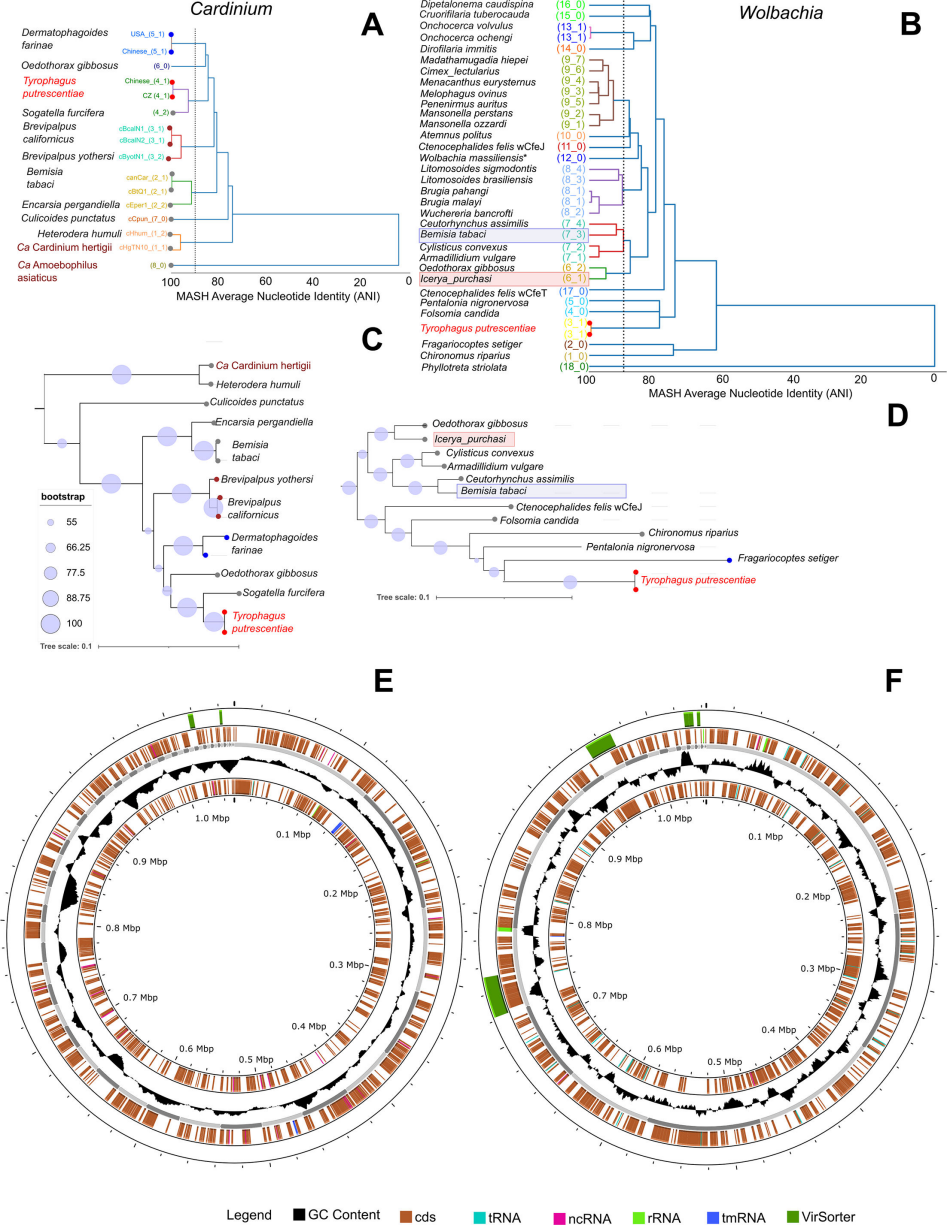
Genomes analyses endosymbiotic intracellular bacteria *Cardinium* (A, C, E) and *Wolbachia* (B, D, F) from the domestic mold mite *T. putrescentiae*. (**A, B**) FastANI analysis using average nucleotide identities (ANI) in dREP (54); (**C, D**) genome similarity analyses based on the open reading frames in M1CR0B1AL1Z3R (55); (**E, F**) genomic assembly visualization in Proksee (56), the virus genes were identified by VirSorter (57) (see Table S2 and S3).

The genome of the *Cardinium* endosymbiont of *T. putrescentiae* (cTput) consists of 55 contigs spanning 1.05 Mbp (Fig. 1; Table 1). This genome was most similar to the previously assembled *Cardinium* from *T. putrescentiae* (GenBank: JANAVR01) (43) (Fig. S1 and S2); however, our assembly resulted in a larger number of predicted genes, 882 versus 769 (Table S2), likely due to the more contiguous assembly. Both assembled genomes lack plasmids, which are present in the *Cardinium* strain from the house dust mite *Dermatophagoides farinae* (*cDfar*) (43) and the parasitoid wasp *Encarsia suzannae* (cEper) (14). The newly sequenced *cTput* strain forms a sister group *to Cardinium* from the planthopper *S. furcifera* (15), based on the analysis of average nucleotide identity (ANI) using dREP (54) (Fig. 1). These results were also supported by a phylogenetic analysis using M1CR0B1AL1Z3R (55) (Fig. 1). Both novel and previously assembled *Cardinium* genomes share 375 (73%) of the predicted KEGG proteins (Fig. S3). The predicted KEGG proteins formed 10 complete modules, including the lipoic acid biosynthesis module in our *cTput* assembly.

**TABLE 1:** Characteristics of select *Wolbachia* and *Cardinium* genomes and *T. putrescentiae* transcriptome^*c*^

Genome/trans.	Host	GenBank ID (reference)	Size (bp)	Compl. (%)	Cont. (%)	Cov.	Contigs	GC (%)	CDSs	KEGG	rRNA	tRNA
*cTPut* (genome)	*T. putrescentiae*	JAUEML01 ^*a*^	1,051,907	100	0	3,541	55	38.9	882	498	3	34
	*T. putrescentiae*	JANAVR01 (42)	914,750				33	39.38	769	446	3	33
	*Sogatella furcifera*	NZ_CP022339 (15)	1,103,593				1	39.23	897	479	3	35
	*Oedothorax gibbosus*	NZ_OW441264 (58)	1,137,202				1	36.70	1,046	623	3	35
*wTPut* (genome)	*T. putrescentiae*	JAUEMM01	1,043,441	100	0	2,118	26	34.50	974	532	3	33
	*T. putrescentiae*	GIJY01000000 (44)	910,457				280	35.10	686	404	0	27
	*Fragariocoptes setiger*	JAHRAF01 (50)	1,082,514				30	31.20	1,101	573	3	39
	*Pentalonia nigronervosa*	NZ_JACVWV01 (51)	1,457,187				182	34.10	1,243	566	3	36
*Tyrophagus putrescentiae*		JBBPFL01 ^*a*^ ^,*b*^	114,502,572	88	—	—	10,330	45.52	13,702	5,841	—	—

^*a*^ This study.

^*b*^ Transcriptome-based annotation based on deposited genome, completeness according to BUSCO for arachnida data set.

^*c*^ —, not determined; Compl., completeness; Cont., contamination.

The genome of the *Wolbachia* endosymbiont of *T. putrescentiae* (wTput) (JAUEMM01) (Fig. 1) consists of 26 contigs spanning 1.04 Mbp (Table 1). This assembly was similar to the previous assembly of wTPut (GIJY01) (45) (Fig. S4, S5, and S7), but the number of predicted genes was higher, 974 vs 686 (Table S3). There were small differences in the predicted KEGG proteins; JAUEMM01 contained 126 KEGG proteins not identified in GIJY01 (45), whereas GIJY01 had 12 KEGG proteins not identified in JAUEMM01 (Fig. S6).

wTput clustered with *Wolbachia* from the plant-feeding mite *Fragariocepes setiger* (59), the aphid *Pentalonia nigronervosa* (60), and the nonbiting midge *Chironomus riparius*, as inferred by a M1CR0B1AL1Z3R phylogenetic analysis (55) and an ANI analysis (Fig. 1). Previous analyses identified wTput as belonging to supergroup Q, which is exclusively associated with acariform mites (45). The wTPut genome had six complete KEGG modules; however, both riboflavin and biotin pathways were incomplete.

According to the known sequences (61), we screen *wmk* and *CifB* genes responsible for CI in wTPut using PHMMER. The analysis revealed that helix-turn-helix domain-containing protein (MDN5248470.1) had 40.6% identity to *wmk_4* (WHMT_12930), 51.7% *to wmk_1* (WHMT_12840), and 54.7% to *wmk_3* (WHMT_12920); *wmk_2* was not identified in wTPut. Ankyrin repeat domain-containing protein (MDN5248188) had 31% identity to *wHm-t Hm-oscar* (WHMT_13140). Hypothetical protein (MDN5248450) had 27.1% identity to *CifB* (WP_320157280). Another ankyrin-repeated protein (MDN5248379) showed 37.6% identity to *cifB* of wStri (53). No other matches were found for cifA and cifB genes for *Wolbachia* groups A and B, as reported in the study by Martinez et al. (53). In summary, there is no evidence for these CI proteins in wTPut.

### Genome and transcriptome of *T. putrescentiae*

The general assembly statistics of our *T. putrescentiae* genome (JBBPFL01) are shown in Table 1; Table S4 and S5. The transcriptome contained 5,838 KEGG annotated proteins (Table S3_1 at https://zenodo.org/records/15172873), which formed 66 complete KEGG modules, including pantothenate, tetrahydrobiopterin, molybdenum cofactor, C1-unit interconversion, and heme. The entire metagenome of the mite, which includes both bacterial symbionts and the mite itself, contained 80 complete KEGG modules.

### Gene expression of intracellular symbionts

**Fig 2:**
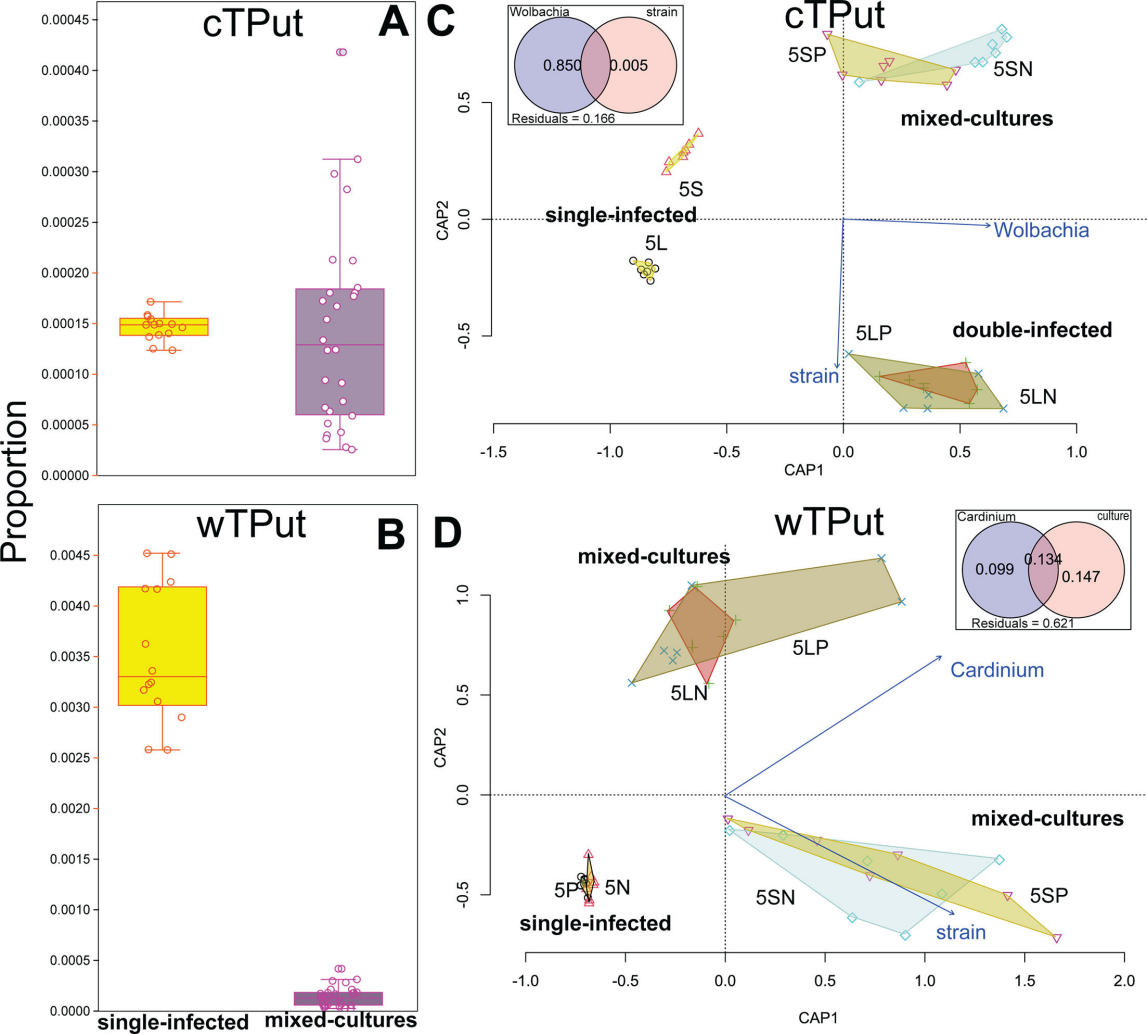
The comparison of single-infected and mixed samples of mite *T. putrescentiae* by *Cardinium* (cTPut) and *Wolbachia* (wTPut). The proportion of transcriptomic reads for cTPut/mite (**A**) and wTPut/mite (**B**). A correlation triplot of distance-based redundancy (dbRDA) models showing interaction among cTPut (**C**) and wTPut (**D**) in different mite cultures: cTPut single-infected (5L, 5S), wTPut single-infected (5P, 5N), and mixed cultures (5LN, 5LP, 5SN, 5SP). The environmental variables were the bacterial strain and the presence of another symbiont. For each model, variable contribution to the model is shown in the Venn diagram insets.

cTPut proportion of transcriptomic reads in single and mixed cultures was similar (Mann–Whitney *U* = 180.5, *P* = 0.689) (Fig. 2A). However, the wTPut proportion decreased 10-fold in mixed cultures (Mann–Whitney *U* = 0, *P* < 0.0001) in comparison to single-infected cultures (Fig. 2) after 42 days of the experiment.

**Fig 3:**
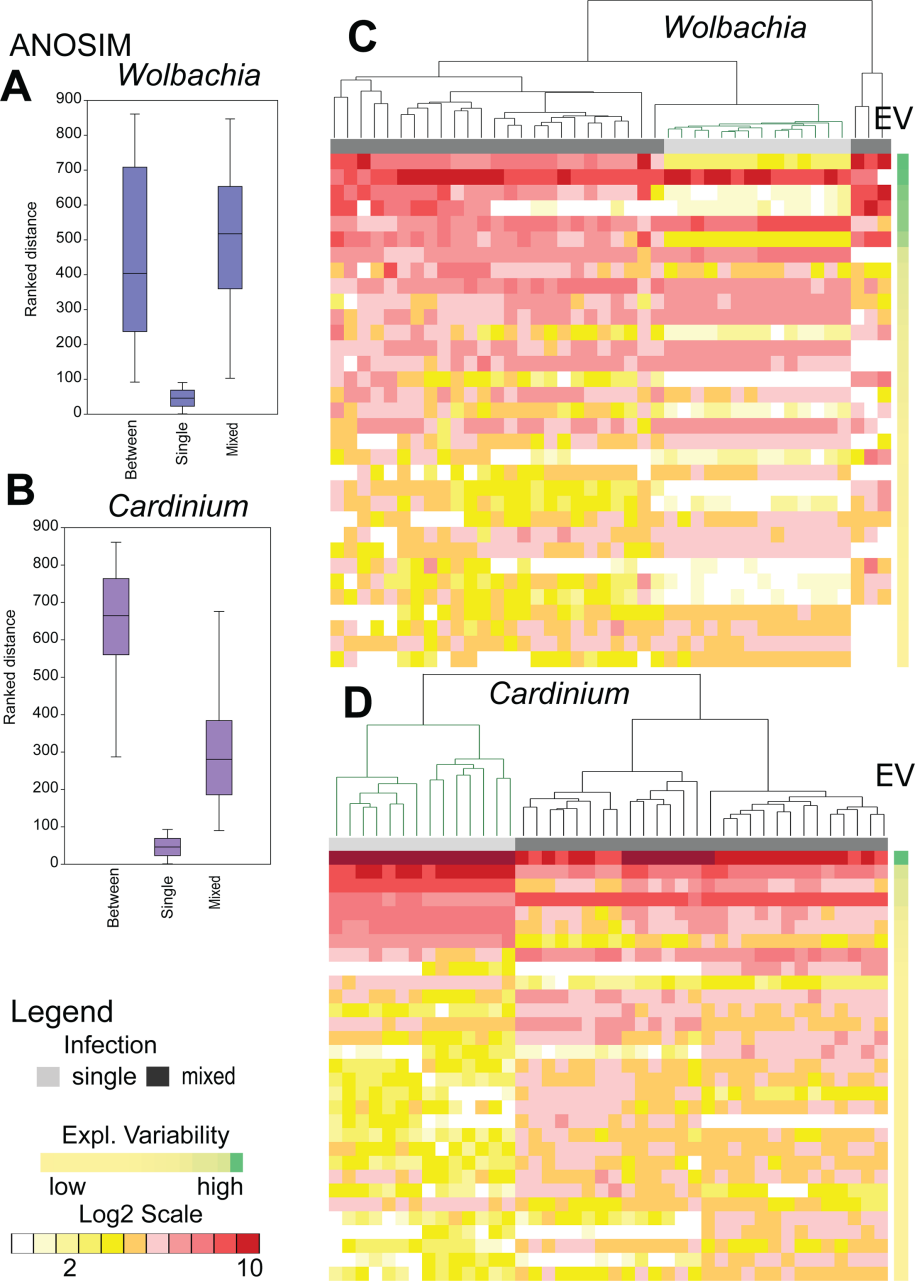
Comparison of *Cardinium* (cTPut) (A, B) and *Wolbachia* (wTPut) (C, D) gene expression in single-infected and mixed cultures of *Tyrophagus putrescentiae*. (**A, B**) Analysis of similarities (ANOSIM) in gene expression between single-infected and mixed samples. (**C, D**) Heatmap analyses of expressed genes (LOG2 transformed) with the highest contribution to dissimilarity, as identified by SIMPER. The explained variability is represented by a color gradient from green (high) to yellow (low). UPGMA clustering of samples was calculated using Bray–Curtis distance, with single-infected samples shown in green. A list of genes is provided in Table S6.

The expression of the predicted *Cardinium* genes differed between single and mixed cultures (ANOSIM: *R* = 0.9573, *P* < 0.001) (Fig. 3; Table S6), a result further supported by dbRDA analyses (Table 2: id 1). For *Cardinium*, an ordination triplot analysis separated variables related to single and mixed cultures along the cap1 axis, whereas the variables related to mite cultures were separated along the cap2 axis (i.e., 5S, 5SN, and 5SP versus 5L, 5LN, and 5LP) (Fig. 2D). For wTPut, no difference in gene expression between single and mixed culture samples was detected (ANOSIM: *R* = 0.08802, *P* = 0.1182) (Fig. 3). These samples (Table S6), however, were still distinguishable by dbRDA analyses (Fig. 2E, Table 2 id 2). dbRDA models (Table 2, ids 1 and 2) showed that cTPut gene expression was more strongly influenced by the presence/absence of wTPut than vice versa, with variability explained being 2-fold greater in the former case. In comparison to single-infected cultures, cTPut causes greater changes in gene expression than wTPut in mixed cultures and strongly influences wTPut gene expression. (Table 2: ids 3 and 4).

**TABLE 2:** Correlation-based gene expression models of two endosymbionts, *Cardinium* (cTPut) and *Wolbachia* (wTPut), and their mite host *T. putrescentiae* (TP) in single-infected and mixed cultures^*a*^^,^^*b*^

Id.	Dependent variable	Independent variable	df	*F*	*R* ^2^
1	cTPut_gene	wTPut presence/absence	3	27.80	0.687
2	wTPut _gene	cTPut presence/absence	3	7.70	0.378
3	cTPut _gene	wTPut _gene	15	2.93	0.786
4	wTput_gene	cTPut _gene	18	2.79	0.848
5	TP_KEGG	cTPut/wTPu presence/absence	3	39.68	0.620
6	TP_KEGG	wTPut _genes (single)	8	32.61	0.981
7	TP_KEGG	wTPut _genes (mixed)	22	99.62	0.997
8	TP_KEGG	cTPut _genes (single)	6	7.15	0.860
9	TP_KEGG	cTPut _genes (mixed)	21	77.40	0.996
10	TP_KEGG	cTPut / wTPut genes	19	66.63	0.994
11	wTPut _genes (single)	TP_KEGG	4	4.03	0.548
12	wTPut _genes (mixed)	TP_KEGG	13	3.14	0.745
13	cTPut _genes (single)	TP_KEGG	4	2.94	0.566
14	cTPut _genes (mixed)	TP_KEGG	11	3.83	0.725

^*a*^ *P*-values were <0.05 for all models; df degree of freedom; F, permutation test value, *R*, variability explained by the tested independent variables.

^*b*^ The models were built using Bray–Curtis distance-based redundancy analyses (dbRDA) and different sets of variables: predicted bacterial genes, mite KEGG genes, and symbiont presence/absence data in single and double-infected mite cultures.

There were differently expressed genes of cTPut and wTPut in single and mixed cultures (Fig. S7). wTPut had 29 upregulated and 69 downregulated genes, cTPut had 12 upregulated and 173 downregulated genes. According to the false-discovery rate (FDR-adjusted *P* < 0.05) (Table S7), 5,837 (95%) predicted KEGG genes in the mite host exhibited differential expression between single- and double-infected cultures (Tables S8 and S9).

### Gene expression analyzed by diversity index

**Fig 4:**
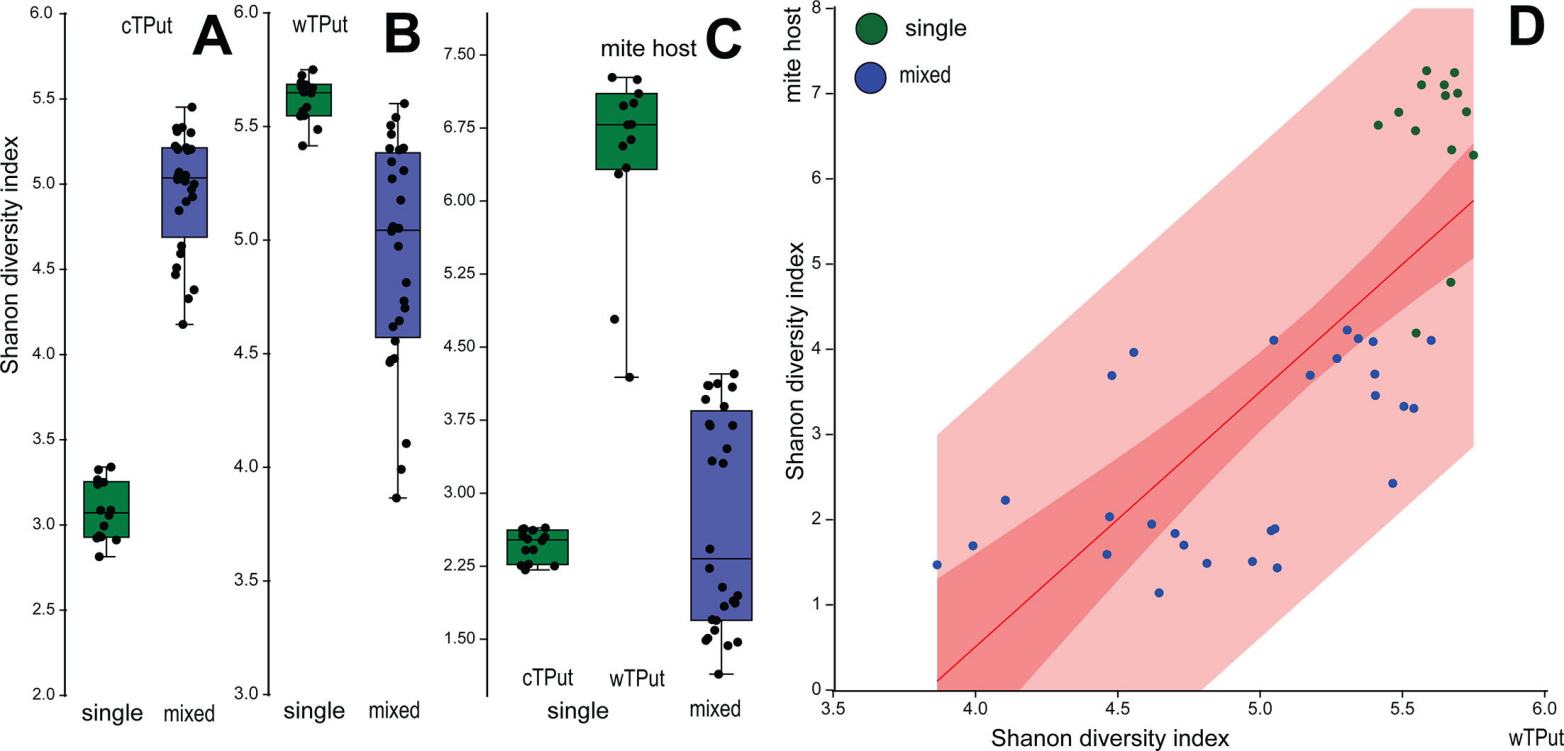
Gene expression diversity of intracellular symbionts and mite (*T. putrescentiae*) host KEGG genes in single-infected and mixed samples using Shannon index. (**A, B,C**) Box and jitter plots for Shannon index in the samples; (A) *Cardinium* (cTPut); (B) *Wolbachia* (wTPut); (C) mite; (**D**) linear regression of Shannon index for *Wolbachia* and its mite host. Dark red is 95% CI, and light red is 95% forecast.

To further compare gene expression diversity among the two endosymbiotic bacteria and their mite host, we used the Shannon diversity index (Fig. 4). In mixed cultures, the gene expression diversity index slightly decreased for wTPut by up to 10%, which was a statistically significant change (Mann–Whitney: 4.92, *P* < 0.001), whereas it increased 1.5-fold for cTPut (Mann–Whitney: 5.22, *P* < 0.001) (Fig. 4AB). For mite KEGG genes, the expression diversity index was 2-fold higher in wTPut single-infected samples compared with both cTPut single-infected samples and mixed samples (Kruskal–Wallis: 30.76, *P* < 0.001) (Fig. 4C). In other words, these data suggest that in double-infected hosts, cTPut increased the numbers of genes involved in the interactions with the host, whereas *wTPut* lost a significant number of interactions. However, overall, wTPut had a higher level of interaction compared with cTPut. In addition, there was a significant positive correlation between wTPut and mite expression diversity indices [ANOVA: *F*_(1,40)_ = 49.218; *P* < 0.001; *R*² =0.55] (Fig. 4D).

### Interaction between *Cardinium* and *Wolbachia* based on gene expression correlation analyses

**Fig 5:**
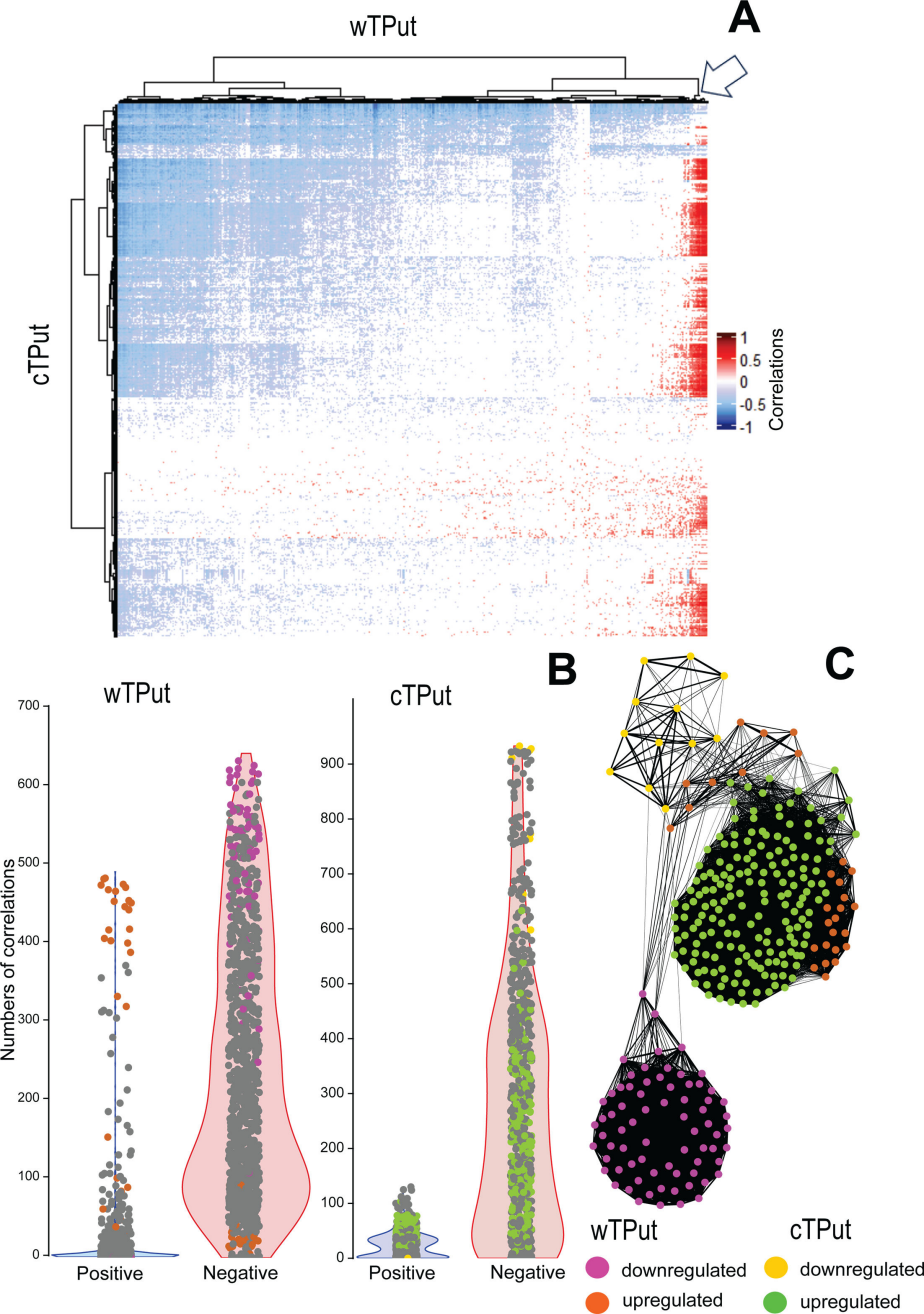
Spearman correlation between *Cardinium* (cTPut) and *Wolbachia* (wTPut) gene expressions in the mixed samples of mite host *T. putrescentiae.* (**A**) Correlation heatmap; (**B**) positive and negative gene expression correlations for the two bacteria; (**C**) Fruchterman–Reignold network plot of Spearman correlations based on Euclidean distance with 75% edge cutoff for upregulated/downregulated genes. The arrow indicates a small cluster of *Wolbachia* cluster 20 genes characterized by high numbers of positive correlations.

The number of negative correlations (Spearman, permutational *P* < 0.05) (Table S3_2 at https://zenodo.org/records/15172873) between cTPut and wTPut gene expressions was 10-fold higher than the number of positive correlations: 239,940 vs 21,444 in mixed culture (Fig. 5). Among the positively correlated genes, there was a small wTPut cluster including 20 genes (Fig. 5, arrow), such as quorum-sensing protein: GTP cyclohydrolase II (MDN5247543), proteins associated with replication and repair processes: DNA recombination protein RmuC (MDN5248228), RNA degradation: ribonuclease J (MDN5247844); ribosome biogenesis: GTPase Der (MDN5247519); and membrane transport: phosphate ABC transporter permease PstA (MDN5247835), pyridoxal phosphate-dependent aminotransferase (MDN5247857), isoprenoid biosynthesis glyoxalase ElbB (MDN5247898), and Tim44/TimA family putative adaptor protein (MDN5248167). However, many of the proteins in this cluster did not have a known function (Tables S2 and S3).

Network analyses revealed that the majority of upregulated wTPut genes interact with cTPut upregulated genes (Fig. 5C). The following wTPut upregulated genes interacted with up-/down-regulated cTPut genes: outer membrane beta-barrel protein (MDN5247899), AsmA-like C-terminal region-containing protein (MDN5248353), alpha/beta fold hydrolase (MDN5248042), and hypothetical proteins, MDN5247862 and MDN5248062. For *Cardinium*, additionally, msbA transporters (MDN5246960 and MDN5247417) had the highest numbers of positive correlations with wTPut-selected genes (Fig. S9 to S11). These correlations included wTPut proteins that were associated with type IV secretion (virD4 and VirB4) and signal recognition particle rfbA.

### Interaction between *Cardinium* and *Wolbachia* and mite host based on gene expression correlation analyses

Gene expression of the mite host was statistically different in single-infected and mixed cultures (KEGG genes: ANOSIM: *R* = 0.5806, *P* < 0.001), particularly, statistically significant differences were between wTPut-infected samples and cTPut-infected or mixed samples (Bonferroni correction, *P* < 0.05) but not between mixed and cTPut-single infected (Fig. S12). This analysis is supported by the dbRDA providing similar results (Table 3, id. 1, Fig. S13). The mite gene expression was most influenced by the symbionts’ gene expression than vice versa (Table 3: ids 2–5 versus 6–10).

**TABLE 3:** Correlation-based gene expression models of *Cardinium* (cTPut), *Wolbachia* (wTPut), and their host *T. putrescentiae* in single-infected and mixed samples^*a*^^,^^*b*^

Id.	Dependent variable	Independent variable	df	*F*	*R* ^2^
1	mite_Pathway_KEGG	Total	8	5.68	0.401
		wTPut _presence	1	11.67	0.135
		cTPut _presence	1	4.18	0.053
		double_infection_presence	1	2.69	0.035
2	wTPut _genes (single)	mite_Pathway_KEGG	11	3.16	0.584
3	wTPut _genes (mixed)	mite_Pathway_KEGG	11	3.16	0.685
4	cTPut _genes (single)	mite_Pathway_KEGG	3	3.09	0.481
5	cTPut _genes (mixed)	mite_Pathway_KEGG	8	4.23	0.641
6	mite_Pathway_KEGG	wTPut _genes (single)	9	36.29	0.988
7	mite_Pathway_KEGG	wTPut _genes (mixed)	19	18.31	0.959
8	mite_Pathway_KEGG	cTPut _genes (single)	19	18.31	0.978
9	mite_Pathway_KEGG	cTPut _genes (mixed)	18	19.75	0.975
10	mite_Pathway_KEGG	cTPut / wTPut _genes	14	19.05	0.954
11	mite_Metabolism_sumKEGG	Total	8	12.87	0.602
12	wTPut _genes (single)	mite_Metabolism_sumKEGG	2	1.58	0.224
13	wTPut _genes (mixed)	mite_Metabolism_sumKEGG	6	2.53	0.420
14	cTPut _genes (single)	mite_Metabolism_sumKEGG	2	1.69	0.235
15	cTPut _genes (mixed)	mite_Metabolism_sumKEGG	4	2.29	0.284
16	mite_Metabolism_sumKEGG	wTPut _genes (single)	8	14.41	0.958
17	mite_Metabolism_sumKEGG	wTPut _genes (mixed)	14	5.55	0.857
18	mite_Metabolism_sumKEGG	cTPut _genes (single)	3	3.39	0.744
19	mite_Metabolism_sumKEGG	cTPut _genes (mixed)	4	6.63	0.919
20	mite_Metabolism_sumKEGG	cTPut/ wTPut _genes	19	7.33	0.946

^*a*^ *P*-values were <0.05 for all models; df degree of freedom; *F*, permutation test value, *R*, variability explained by the tested independent variables.

^*b*^ Our distance-based redundancy models (dbRDA) used robust Aitkinson distances and a set of variables: predicted genes (symbionts), KEGG genes (mites) and the presence/absence of symbionts in single-infected and mixed mite cultures. Selected mite genes were associated with the mite immune and regulatory pathways or metabolism.

**Fig 6:**
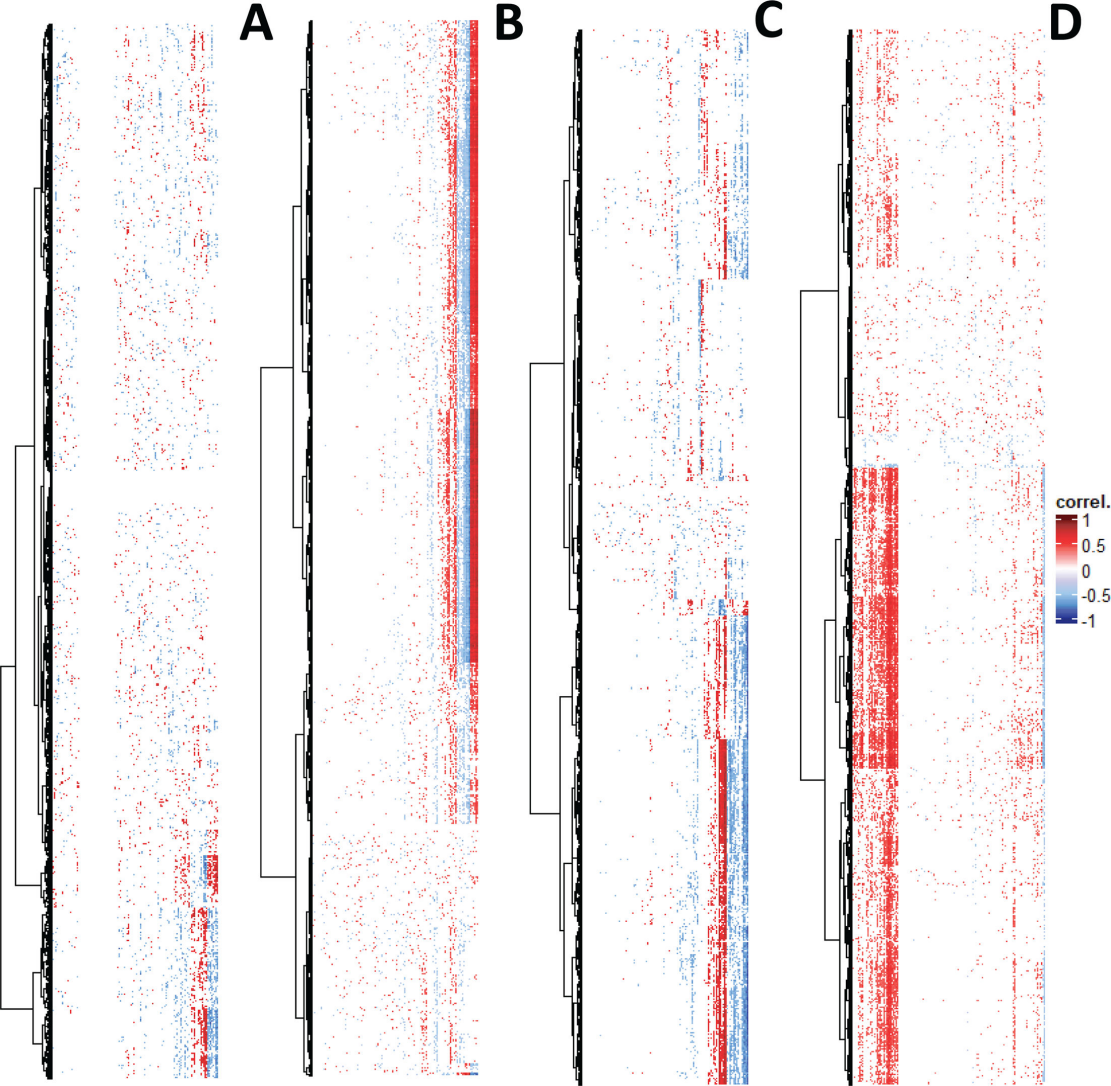
Spearman correlation of gene expression in *Cardinium* (cTPut) (A, B) and *Wolbachia* (wTPut) (C, D) versus the mite host *T. putrescentiae* in single-infected (A, C) and mixed (B, D) cultures. Only correlations with *P* < 0.05 are included. For the mite, only KEGG genes were used. The correlations are expressed as a heatmap, *Cardinium* or *Wolbachia* on *x* axes and mite KEGG genes on *y* axes.

A comparison of the Spearman correlations between the mite-predicted KEGG gene expression and cTPut or wTPut in single-infected and mixed cultures (Table S3_3 at https://zenodo.org/records/15172873) revealed changes in the numbers of correlations (Fig. 6). wTPut had 218,872 positive and 274,741 negative correlations to mites in a single-infected culture. The interaction in mixed cultures was characterized by a 2-fold decrease in positive correlations and a 10-fold decrease in negative correlations with 121,784 positive and 23,654 negative correlations (see Fig. S15 for statistical analyses). cTPut had 114,266 positive and 123,335 negative correlations to mite KEGG genes in single-infected cultures. The number of positive correlations slightly increased to 162,708 in mixed cultures, and the number of negative correlations decreased to 91,634. In summary, the interaction of wTPut and its mite host strongly decreases in mixed cultures.

Among the 5,842 predicted KEGG genes of the mite host (Table S8), 75 genes were identified as outliers (Table S9). Our cluster analyses of gene expression correlations to cTPut and wTPut identified remarkable changes in mite host gene correlations to wTPut in single-infected and mixed cultures (Table S9). For example, cluster 6 included 18 KEGG genes (e.g., clathrin, actin, importin, and ABC transporters) characterized by a strong decrease of negative correlations to wTPut in mixed cultures (Table S9). Clusters 3 and 5 had a high number of positive correlations to wTPut in mixed cultures (i.e., glutamate receptor, serpin, cathepsin L, and ubiquitin-associated enzymes) (Table S9). Clusters 1 and 2 had increased positive or negative correlations to both cTPut and wTPut in mixed cultures (Table S9). These genes were associated with ribosomes and metabolism. Cluster 4 had an increase in correlation between mite and cTPut in mixed cultures and included genes associated with metabolism and genetic information processing (Table S9).

The wTPut outliers included 74 genes with a high number of positive/negative correlations to the mite host gene expression in single-infected and mixed samples (Table S10), which were clustered in five groups. Group 1 included genes whose expression was not affected by single-infection and mixed cultures and had high numbers of positive correlations to the mite expression in both cultures, for example, chaperonins (MDN5248116, MDN5248117), porin (MDN5247556), and P44/Msp2 family outer membrane protein (MDN5247531) (Table S10). Genes in the remaining groups had a response to double infection. Group 2 represents genes showing an increase of positive correlations in mixed cultures, for example, transporters (MDN5248134, MDN5247677, and MDN5247575) and type IV secretion system proteins VirB8 (MDN5247542) (Table S10). In contrast, group 3 had an increase of negative correlations in mixed cultures, for example, cell division protein ZapA (MDN5248238), pyridoxine 5'-phosphate synthase (MDN5248256), and pyridoxal phosphate-dependent aminotransferase (MDN5247857). Ribosomal proteins (MDN5248018, MDN5247785, MDN5247599, and MDN5248013) formed a group with a high number of positive correlations in single-infected samples, but not mixed cultures (Table S10). Although metal and lipid transferases (WP_088414456, MDN5248113, MDN5247891, and MDN5247743) had a high number of negative correlations in single-infected cultures, this effect disappeared in mixed cultures. For wTPut, a single gene encoding actin-binding protein MDN5248151 was identified with a 42.7% similarity to actin-binding proteins of *Wolbachia* symbiont from *Drosophila melanogaster* WD0830 (GenBank AAS14517) (62) (Table S11) (Fig. S16). This protein exhibited a higher number of positive correlations above the medians in both cultures.

cTPut outliers included 37 genes grouped in three clusters (Table S10). In contrast, to wTPut, cTPut had only one cluster with genes having a high number of both positive/negative correlations to mite gene expression in single-infected samples (but not in mixed samples), for example, chaperonin (WP_260536873) and cold-shock protein (WP_114910160) (Table S10). Like *Wolbachia*, there were two clusters with increased positive or negative correlations in mixed cultures; these clusters included Colicin import membrane protein (MDN5247505), sporulation initiation inhibitor Soj (MDN5247422), and transposases (MDN5247343, MDN5247491, and MDN5247508) with a high number of positive correlations to the mite genes (Table S10), whereas the following protein-encoding genes had a high number of negative correlations: cytosol aminopeptidase (WP_260536811), chromosome partitioning protein (WP_260537191), and aminotransferase (WP_260537063) (Table S10).

### The effect of *Cardinium* and *Wolbachia* on the mite immune and regulatory pathways and metabolism in single-infected and mixed cultures

We built partial distance-based redundancy analyses (dbRDA) models to compare the effect of wTPut or cTPut gene expression to predict mite KEGG-annotated genes of select pathways in single- and double-infected samples (Tables S12 and S13). These models enable comparison of the effect of explanatory variables, such as cTPut and wTPu*t* gene expressions, to the mite gene expression in terms of explained variability (Table 4). In single-infected cultures, wTPut had more interactions (explained variability: 0.84–0.99) than cTPut (0.47–0.94) (Table 4). Mixed samples had more complex interactions, with the number of explanatory genes (df values) increasing two or three times (Table S13), and cTPut establishing more interactions (explained variability: 0.80–0.98) (Table 4). The mite gene expression response was grouped into six clusters. Clusters 1 and 5 had a high level of control of the mite pathways by both symbionts in single-infected and mixed cultures (Table 4). The differences between the two clusters were due to cTPut in single-infected cultures with a low effect on cluster 1 and a higher effect on cluster 5 (Table 4). In mixed cultures, wTPut did not lose its influence on mite pathways, such as Sphingolipid signaling pathway, TOLL, and NOD_like signaling pathway (clusters 1) and Phagosome, TGF-beta (cluster 5). Clusters 2 and 6 had a low effect by both symbionts in single-infected cultures; however, in mixed cultures, cTPut or both symbionts had a higher effect on the mite pathways, for example, Wnt signaling pathway, mTOR, and bacterial invasion of epithelial cells (cluster 2) and Apelin and TNF (cluster 6) (Table 4). For cluster 4, in double-infected cultures, several pathways responded to cTPut (Table 4), for example, regulation of actin cytoskeleton, ubiquitin-mediated signal pathway, and apoptosis. Cluster 3 included pathways with a low effect of cTPut in single-infected cultures, but a higher effect of cTPut in mixed cultures, for example, endocytosis (Table 4).

**TABLE 4:** The effect of *Wolbachia* (wTPut) and *Cardinium* (cTPut) symbionts on select mite immune and regulatory pathways expressed as the explained variability of models R^*a*^^,^^*b*^

Item	Cluster	Silhouette	wTPut S	cTPu S	wTPut M	cTPu M	Both
Sphingolipid	1	0.511	0.968	0.830	0.970	0.958	0.970
JAK_STAK	1	0.433	0.980	0.808	0.987	0.963	0.985
TOLL	1	0.401	0.991	0.823	0.947	0.941	0.964
Notch	1	0.352	0.974	0.856	0.955	0.964	0.983
TOLL_IMD	1	0.260	0.982	0.862	0.995	0.981	0.980
Hedgehog	1	0.203	0.951	0.823	0.935	0.960	0.966
NF_kappa	1	0.201	0.988	0.864	0.968	0.948	0.980
NOD_like	1	0.005	0.952	0.855	0.945	0.946	0.927
PI3K_Akt	2	0.129	0.845	0.769	0.855	0.878	0.883
mTOR	2	−0.009	0.848	0.686	0.862	0.911	0.913
Wnt	2	−0.060	0.916	0.773	0.938	0.910	0.871
Oocyte meiosis	2	−0.118	0.876	0.726	0.928	0.939	0.943
Phospholipase D	2	−0.123	0.910	0.808	0.873	0.808	0.942
Mitophagy	2	−0.208	0.909	0.742	0.916	0.926	0.927
Autophagy	2	−0.217	0.910	0.749	0.907	0.943	0.918
Bacterial invasion of EC	2	−0.221	0.897	0.746	0.911	0.923	0.971
Peroxisome	2	−0.271	0.923	0.684	0.899	0.878	0.906
cAM	3	0.058	0.873	0.487	0.879	0.918	0.897
Endocytosis	3	0.041	0.968	0.474	0.845	0.866	0.901
Hippo	3	−0.388	0.933	0.566	0.907	0.912	0.958
Ras	4	0.424	0.960	0.703	0.913	0.930	0.958
ErbB	4	0.336	0.949	0.669	0.921	0.953	0.950
Regulation of actin cytoskeleton	4	0.310	0.951	0.729	0.923	0.928	0.926
Ubiquitin	4	0.291	0.931	0.718	0.891	0.953	0.948
Apoptosis	4	0.288	0.945	0.749	0.919	0.956	0.954
Proteasome	4	0.285	0.949	0.728	0.936	0.915	0.948
Rap1	4	0.272	0.955	0.738	0.926	0.972	0.921
Calcium	4	0.251	0.947	0.690	0.866	0.911	0.932
MAPK	4	0.226	0.973	0.668	0.840	0.922	0.930
Insulin	4	0.125	0.940	0.699	0.882	0.888	0.929
p53	4	0.008	0.939	0.773	0.928	0.927	0.960
Phosphatidylinositol	5	0.446	0.972	0.940	0.945	0.919	0.948
Phagosome	5	0.415	0.976	0.930	0.945	0.935	0.967
Lysozyme	5	0.218	0.966	0.911	0.929	0.895	0.926
HIF_1	5	0.217	0.963	0.928	0.964	0.954	0.978
TGF_beta	5	−0.240	0.960	0.881	0.960	0.932	0.968
AMPK	6	0.245	0.882	0.821	0.943	0.945	0.943
Apelin	6	0.133	0.894	0.845	0.956	0.924	0.976
cGMP_PKG	6	0.094	0.880	0.881	0.940	0.915	0.941
FoxO	6	0.091	0.899	0.858	0.893	0.908	0.937
TNF	6	−0.078	0.894	0.796	0.945	0.962	0.977

^*a*^ Partial dbRDA models were built for every pathway using mite immune and regulatory pathway expression in single infected (S) or mixed (M) cultures. The independent variables were gene expression I f following data sets (i) wTPut, (ii) cTPut, or (iii) both wTPut and cTPut. The models are described in Table S12.

^*b*^ The K means clustering algorithm was used for *R* values (WGGS = 0.130, *F* = 4.320, Var% = 81.203, Av. Silh = 0.130).

In wTPut single-infected samples, mites had a higher fold expression in metabolic pathways (Table S14) than those infected with cTPut (ANOSIM *R* = 0.654, *P* < 0.001). The same pattern was recovered by dbRDA analyses (Table 3: ids 16 and 18). Among these metabolic pathways, the Krebs cycle, beta-oxidation, and fatty acid biosynthesis had 15–20 times higher gene expression (Table S14) in wTPut-infected samples compared with cTPut-infected samples. Mixed cultures had different gene expression profiles compared with the single-infected samples (ANOSIM: cTPut *R* = 0.136, *P* = 0.02; wTPut *R* = 0.484, *P* < 0.001). However, for cTPut, the differences did not exceed a 4-fold change. In the mites from wTPut-infected and mixed cultures, the Krebs cycle, beta-oxidation, and fatty acid biosynthesis showed a decrease in double-infected samples, whereas the remaining pathways remained unchanged (Table S15).

## DISCUSSION

The common domestic mold mite, *T. putrescentiae*, harbors two major endosymbiotic bacteria, *Cardinium* and *Wolbachia*, but these bacteria almost never occur together in natural populations or long-term laboratory cultures, suggesting potential antagonistic interactions. Our phylogenomic analyses revealed that *Cardinium* from *T. putrescentiae* (cTPut) is related to *Cardinium* found in the planthopper *S. furcifera* (cSFur) (15). We also detected the absence of the two plasmids previously identified in *Cardinium* associated with the pyroglyphid house dust mite, *D. farinae* (cDFar) (cDFar) (43). cTPut forms a single, broadly distributed strain of *Cardinium* associated with *T. putrescentiae*, which has been detected so far in China (43) and Europe using 16S rRNA data (39, 63). cTPut has a biosynthetic pathway for lipoic acid that could supply lipoate to its host, *T. putrescentiae*, similar to the cDFar symbiont of *D. farinae* (64). However, cTPut cannot provide biotin to its acarine host.

Another endosymbiotic bacterium of *T. putrescentiae*, *Wolbachia* is part of *Wolbachia* supergroup Q, representing one of the earliest divergences in this bacterial genus; this supergroup is mostly or exclusively specific to acariform mites (45). The next basal *Wolbachia* divergence, supergroup E, contains symbionts of springtails and oribatid mites (65). Studies reported that *Wolbachia* from planthoppers, such as *Laodelphax striatellus* and *Nilaparvata lugens*, can provide biotin and riboflavin to their insect hosts, acting like mutualists (66). However, genomic analyses showed that the provisioning of vitamins and nutrients is more complex than previously thought (67). Our study found that wTPut lacks complete biotin and riboflavin pathways, indicating that it cannot provide any new nutrients to the mite host and likely functions as a nutritive parasite. *Wolbachia* from the aphid *P. nigronervosa* (supergroup M) (68) also does not provide any nutrients to its host (69) but provides protective benefits under certain conditions (68).

Here, we characterize these two bacteria through genomic sequencing, gene and pathway annotation, and gene expression analyses in single-infected and mixed cultures (containing double-infected mite individuals) to find possible clues about the nature of interactions between these bacteria and their host. Although the molecular mechanisms underlying the interactions between the two bacterial endosymbionts and their mite host remain poorly understood, we leverage the correlative data to reveal gene expression patterns. Our experiments show that cultures infected with *Cardinium* had different gene expressions between single- and double-infected samples, whereas no such effect was observed for *Wolbachia*. Based on the population-level meta-transcriptome samples, the relative numbers of transcriptomic reads of wTPut 10-fold decreased in mixed cultures, whereas no changes were observed for cTPut in mixed versus single-infected cultures. This agrees with previous observations, when qPCR using specific primers indicated a 10-fold decrease in wTPut after 21 days of experiment (46). The decrease of wTPut proportion on population-level samples can be explained as (i) a decrease in *Wolbachia*-infected individuals and/or (ii) a decrease in the *Wolbachia* titer in individuals. However, this observation contrasts with data from double-infected thrips *P. kellyanus*, *in* which *Wolbachia* was 20 times more abundant than *Cardinium*.

Interestingly, removing *Wolbachia* did not affect the density of *Cardinium* (33), suggesting that there is no competition between the two bacteria within the trip host. The density of *Wolbachia* from *Nasonia vitripennis* was influenced by temperature, host development stage, and interaction with WO phage (70). Overall, our data suggest that *Cardinium*-infected hosts eliminate *Wolbachia*-infected hosts through cytoplasmic incompatibility (CI) or additive benefits, such as a shortened life cycle or higher reproductive efficiency. Alternatively, *Cardinium* has a general pathogen-blocking effect, as was observed in *Oedothorax gibbosus* previously (71). This aspect should be investigated further in other systems involving mites and ticks transmitting pathogens.

Our analyses show that wTPut possibly directly interacts with cTPut as their expression levels of several genes correlate, such as genes encoding quorum-sensing proteins, ABC transporter, and secretory proteins. In mixed cultures, competition for resources between cTPut and wTPut may occur. One potential area of competition lies in the acquisition of lipopolysaccharide (LPS) precursors, vital components for the outer membrane of gram-negative bacteria. Notably, cTPut possesses a lipopolysaccharide export system (lptB, lptF, and lptG) for core-LPS transport, whereas no such transporters were identified in wTPut. This asymmetry in LPS transport capabilities suggests a competitive advantage for *Cardinium* in acquiring LPS precursors within a shared environment. A parallel exists in cooperation for LPS synthesis in mealybugs *Cinara cedri* and its co-obligate symbionts (72). This competition could serve as a regulatory mechanism for both bacteria during co-infection.

Given the documented manipulation of host immunity and regulation by *Wolbachia* and *Cardinium* (2, 7, 73–82), our observed correlations between cTPut and wTPut gene expression with mite immune, regulatory, and metabolic pathways are unsurprising. Notably, our gene expression analysis suggests that wTPut may establish a stronger interaction with the mite’s immune and regulatory pathways compared with cTPut, even in single-infected cultures. This finding warrants further investigation into the specific mechanisms employed by *Wolbachia* to potentially manipulate the mite host. Our data, along with previous observations in *S. furcifera* (35), suggest that competition between symbionts like cTPut and wTPut in mixed cultures of mites primarily occurs through the manipulation of host pathways. In single infections of *S. furcifera*, *Cardinium* increased metabolic activity, whereas *Wolbachia* appeared to suppress it in double-infected individuals (35). Similarly, in our study, wTPut lost control over the mite’s immune, regulatory, and metabolic pathways in mixed cultures of *T. putrescentiae*, whereas cTPut responded by increasing its interaction with the host, potentially through altered gene expression. This pattern aligns with observations in silkworm cell cultures (36), where *Wolbachia* infection had minimal impact on gene expression or immune responses, whereas *Cardinium* triggered the expression of immune-related genes. These findings collectively suggest that indirect manipulation of the host, rather than direct competition between the bacteria, is a more likely strategy for these symbionts to compete in co-infected environments. Our conclusions are further supported by both correlation-based analyses and analyses using the Shannon diversity index, a measure of community complexity commonly used in ecology (83). Recent studies have successfully applied this index to analyze gene expression data (84).

In single-infected cultures, wTPut showed overexpression of genes associated with metabolic pathways like the Krebs cycle, beta-oxidation, and fatty acid biosynthesis, compared with cTPut in mites from mixed cultures. The stimulation of metabolism provides no beneficial effect on wTPut in single-infected cultures, but cTPut in single-infected cultures decreased its expression in comparison to asymbiotic cultures and wTPut single-infected. The same effect was in mixed mite cultures. This downregulation of metabolic pathways in double-infected mites likely contributes to the previously observed fitness costs of competition, such as decreased population growth in *T. putrescentiae* (46) and reduced egg development in *S. furcifera* (85). These findings support the hypothesis that competition between these symbionts occurs likely through manipulation of host pathways, ultimately impacting the mite’s fitness.

Based on mite fresh weight and sample size, the estimated numbers of double-infected mites ranged from 750 to 1,000 individuals per sample in the mixed culture. Our study provides evidence for competition between cTPut and wTPut in mixed cultures based on mite population-level samples. The estimated numbers of mites ranged from 7,500 to 10,000 individuals per sample, including 750–1,000 double-infected individuals. Our study has several limitations: (i) population-level samples, which prevented the separate analysis of double-infected individuals; (ii) the potential influence of interactions with other endosymbiotic bacteria (i.e., gut symbionts *Bartonella*-like, *Sodalis*-like and *Solitalea*-like); and (iii) the use of correlation-based analyses, which may not accurately reflect true causation. Further research using single-specimen gene expression data is needed to address these limitations; however, this approach is technically challenging due to the small amounts of total RNA in mites.

Despite these limitations, our study establishes a novel model for investigating interactions between *Wolbachia* and *Cardinium*. This system is particularly interesting because wTPut belongs to supergroup Q, which is distinct from the typical *Wolbachia* supergroups A and B. Since *T. putrescentiae* is a medically relevant mite due to its role in allergen production (86), understanding how symbionts like *Cardinium* and *Wolbachia* manipulate the mite’s physiology could be crucial for future research into allergen exposure (44, 63). Previous studies have already shown variations in allergen production among different *T. putrescentiae* populations (63, 87). These findings suggest that intracellular bacteria may play a role in allergen production, potentially impacting human health and stored product contamination. Furthermore, our data suggest that *Cardinium* may have a general pathogen-blocking effect, which warrants further investigation in other systems involving mites and ticks that transmit pathogens.

## MATERIALS AND METHODS

### Single-infected mite cultures

For our experiments, four cultures of *T. putrescentiae* that were infected with either *Cardinium* or *Wolbachia* were used: 5L, collected by E. Zdarkova in grain, Bustehrad, Czechia, in 1996, infected by *Cardinium*; 5S, collected by A. Sala in a food producing factory, Cesena, Italy, in 2013, infected by *Cardinium*; 5N, collected by J. Hubert in a food producing factory, St. Louis, MO, USA, in 2007, infected by *Wolbachia*; and 5P, laboratory culture maintained by T. W. Phillips, KSU, Manhattan, KS, USA, obtained in 2014, infected by *Wolbachia*. The cultures were maintained at the Czech Agrifood Research Center (CARC; Crop Research Institute until 2024) in Prague, Czechia. The mites were kept in IWAKI 70 mL tissue culture flasks with a surface area of 25 cm^2^. These flasks were placed in Secador desiccators by Bel-Art Products, which maintained a relative humidity of 85% through a saturated KCl solution. The desiccators were kept in darkness and under controlled conditions of humidity (75% RH) and temperature (25 ± 1°C). The mites were fed a diet called SPMd, which consisted of wheat germ and Mauripan-dried yeast extract (*Saccharomyces cerevisiae*) in a 10:1 (wt/wt) proportion. The diet was mill-powdered, sieved (mesh size, 500 µm), and heated to 70°C for 0.5 h before being fed to the mites.

### Mixed cultures of mites originated from single-infected parental cultures

To obtain mixed cultures, we transferred 10 unsexed adults from a *Cardinium*-infected culture (5L or 5S) and another 10 from a *Wolbachia*-infected culture (5N or 5P) into a new flask. We prepared double-infected cultures 5LN, 5LP, 5SN, and 5SP in each flask with 20 mites in every combination. Previous analyses showed that such cultures contained cTPut-infected individuals, wTPut-infected individuals, and double-infected cTPut + wTPut individuals (46). For transcriptome analyses, we prepared seven replicates = flasks on 0.3 g of SPM. The flasks were stored in desiccators for 42 days under the same conditions used for mite rearing. We designed our experiments based on the fact that *T. putrescentiae* completes its life cycle from egg to adult in 9.4 days (49). No evidence of *Wolbachia/Cardinium*-induced feminization, male killing, or skewed sex ratios was observed in our mite populations. The sex ratio remained close to 1:1 in both single- and mixed-infected mite cultures. Furthermore, there were no noticeable changes in mite lifespan that could confound the interpretation of our results. Therefore, the demographic stability of the host populations supports the validity of our gene expression data, which was unaffected by potential sex ratio distortions or lifespan variations.

### RNA and DNA extraction

Both single- and double-infected cultures were harvested after 42 days of cultivation for transcriptome and genome analyses. This corresponds to a mite culture with exponential growth, which is commonly used for allergenic extracts (88, 89). The preparation of transcriptome and genome samples was described previously (90). Live adult mites were collected from the surfaces of the flasks and plugs, using a brush, and placed into sterile Eppendorf tubes, whereas the eggs and juveniles were concentrated in the diet on the bottom of the flasks. The samples were weighed on a microbalance to obtain 30–40 mg of fresh weight. The fresh weight of mites was estimated using weighted bottles. The mites (between 200 and 350 individuals) were added into the bottle and weighed using a Mettler-Toledo microbalance to two digital points. Then, the mites were killed in 80% ethanol and counted. The weight of fresh mite is 3.61 ± 0.87 µg (mean ± standard deviation, *N* = 12). After 42 days, 10% of the mites were double-infected (48). This suggests that the sample size ranged from 7,500 to 10,000 mites, including 750 to 1,000 double-infected individuals per sample.

All subsequent procedures with RNA isolation were carried out on ice. Mites were surface cleaned using the following method: they were placed in a 100% ethanol solution and vortexed for 5 seconds, followed by centrifugation at 13,000 × *g* for 1 min. Then, the supernatant was replaced with a 1:10 solution of bleach (5% sodium hypochlorite) and ddH_2_O, vortexed for 5 s, and centrifuged at 13,000 × *g* for 2 min. Then, the cleaning solution was removed, the mites were washed in ddH_2_O, and the previous step was repeated. The mite samples were then homogenized in a glass tissue grinder (Kavalier glass, Prague, Czechia) in 500 µL of lysis buffer for 30 s.

RNA extraction was performed using the NucleoSpin RNA kit (catalog no. 740984.50; Macherey-Nagel, Duren, Germany) with the following modifications: homogenized samples were centrifuged at 2,000  ×  *g* for 3 s, and DNA was degraded by DNase I at 37°C according to the manufacturer’s protocol (Riboclear plus, catalog no. 313-50; GeneAll, Lisbon, Portugal). RNA quality was evaluated using a NanoDrop instrument (NanoDrop One; Thermo Scientific, Waltham, MA, USA) and an Agilent 2100 Bioanalyzer (Agilent Technologies, Santa Clara, CA, USA).

For DNA extraction, homogenates were incubated overnight with 20 µL of proteinase K at 56°C. DNA was then extracted using the QIAamp DNA Micro Kit (Qiagen, Hilden, Germany, cat. No. 56304) following the manufacturer’s protocol for tissue samples. The extracted DNA samples were quantified using a Qubit dsDNA HS Assay Kit (Life Technologies). The samples were then transported on dry ice to the MrDNA laboratory (Shallowater, TX, USA) for downstream processing and sequencing.

### Genome and transcriptome sequencing and processing

RNA samples were adjusted to a volume of 30 µL, and the total RNA concentration was determined using the Qubit RNA Assay Kit by Life Technologies. To remove ribosomal RNA, 700 ng RNA samples were treated with the Ribo-Zero Plus rRNA Depletion Kit from Illumina. The rRNA-depleted samples were quantified, with RNA concentration ranging from 9.7 to 13.7 ng/µL, and used for library preparation with the KAPA mRNA HyperPrep Kits from Roche, following the manufacturer’s instructions. After library preparation, the final concentration of all libraries ranged from 49.4 to 74.20 ng/µL, and the average library size was determined using the Agilent 2100 Bioanalyzer from Agilent Technologies. The libraries were then pooled in equimolar ratios of 0.6 nM and sequenced in paired-end mode for 500 cycles with the NovaSeq 6000 system from Illumina. These reads were deposited in GenBank as projects PRJNA493156 and PRJNA990474 (Table S1).

The concentration of DNA in the original sample was around 90 ng/µL, as measured with the Qubit dsDNA HS Assay Kit from Life Technologies. The quality of the DNA was determined using the NanoDrop 2000 from Thermo Fisher Scientific; DNA cleaning was performed using the DNeasy PowerClean Pro Cleanup Kit from Qiagen (final absorbance 260/280 ranged from 2 to 2.2). The sample was then sheared using the Covaris G-tube from Covaris Inc. The average size of the sheared library was determined using the Agilent 2100 Bioanalyzer from Agilent Technologies. 500 ng of the sheared DNA was used with the SMRTbell Express Template Prep Kit 2.0 from Pacific Biosciences. During library preparation, the sample underwent DNA damage and end repair, and barcode adapter ligation. After library preparation, the final library concentration (about 33 90 ng/µL) was measured using the Qubit dsDNA HS Assay Kit from ThermoFisher Scientific. Additionally, the average library size (8,107 bp) was determined using the Agilent 2100 Bioanalyzer from Agilent Technologies. Finally, the library was sequenced using the 10 h movie time on the PacBio Sequel from Pacific Biosciences.

The libraries for Illumina sequencing were prepared using the Illumina DNA Prep Fragmentation library preparation kit, following the manufacturer’s guidelines. For each library preparation, we used 50 ng of DNA. DNA fragmentation and adapter ligation were performed simultaneously, followed by a limited-cycle PCR to add unique indices to the sample. Afterward, the libraries were pooled in equimolar ratios of 0.6 nM and sequenced paired-end for 500 cycles using the NovaSeq 6000 system from Illumina. The reads were deposited in GenBank as project PRJNA988410 (Table S1).

Methods for read processing, genome and transcriptome assembly, and annotation were previously described (90). Illumina reads were trimmed with Trim Galore (91) and analyzed using fastQC (92). The reads were then mapped onto reference data sets using Bowtie2 (93, 94), and Minimap2 (95) was used for long reads. The reference data sets included *Cardinium* and *Wolbachia* genomes, as well as astigmatid mite genomes and transcriptomes available in GenBank. The mapped short Illumina reads and long PacBio reads were *de novo* assembled in hybrid SPADES v3.14 (96, 97). The assembled genome was polished using Pilon (98).

Bacterial sequences were annotated by Prokka (99) using DFAST (100) on a web server, and predicted proteins were identified by KEGG using GhostKoala (101). Bacterial genome annotations were done in Prokka v1.14.6 (99) and visualized in Proksee (56).

The genome and transcriptome of *T. putrescentiae* were annotated using Funannotate 1.8.15 (102) on the Galaxy server (103). Predicted proteins were assigned to KEGG categories, and metabolic pathways were identified using a KEGG mapper (104). Additional analysis was performed using the EggNOG Mapper (105). The presence of predicted KEGG proteins was compared in assemblies and related KEGG proteins using Venn diagrams (106, 107) (package ggVennDiagram in R version 4.3.1) (108).

Gene expression analyses of the bacterial symbionts were performed in CLC Workbench 22 (Qiagen, Venlo, Netherlands). The transcriptome data were analyzed as standardized data (Table S2 and S3) by recalculating the samples with the lowest number of reads, as previously described for amplicon sequencing analyses (109). For the proportions of cTPut/mite and wTPut/mite, the unstandardized read counts were used and then standardized as proportions.

### Phylogenomic and molecular identification

Genome-level taxonomic classification analyses of our *Cardinium* and *Wolbachia* were conducted using the MASH algorithm (110) in dRep (54) on the Galaxy server (103) and GenBank data. Selection of open reading frames (ORFs), identification of orthologous groups, alignment of orthologous sequences (111, 112), and inference of a maximum likelihood phylogenetic tree using RAxML with 100 bootstrap replicates (113) were performed in M1CR0B1AL1Z3R (55). Phylograms were finalized in iTOL v.6 (114).

### Statistical analyses

The effect of single- and double-infected cultures on bacterial and genome expression (proportion cTPut/mite and wTPut/mite) was analyzed using the nonparametric Mann–Whitney test and Kruskal–Wallis test in PAST 4 (115). We adopted a protocol used for analyses of alpha, beta, and gamma diversity measures of bacterial OTUs (109) for our genome expression analyses. Our alpha diversity analyses included the application of the Shannon diversity index (116, 117) calculated in PAST to compare the gene expression of *Cardinium*, *Wolbachia,* and mite in single-infected and mixed samples. Our beta diversity analyses included two types of correlation analyses to correlate the expression of bacterial and mite genes. First, distance-based redundancy analyses (dbRDA) were used to test the correlation between the expression of the predicted genes and selected factors: single- and double-infected cultures, presence/absence of wTPut and cTPut, and gene expression data. The analyses were performed using the vegan package (118) in R version 4.3.1 and using ANOSIM in PAST (Bray–Curtis distance). In the dbRDA models, we compared data sets as “dependent-gene expression” and “independent-environmental variables” (119). We also did analyses using gene expression data from bacteria or mites as dependent and independent variables interchangeably (64). We calculated dbRDA models using Bray–Curtis distance for standardized data or Robust Atkinson distance for unstandardized data. To identify the variables with the strongest impact on our model, we used a forward selection approach implemented through the “ordistep” function. This function is specifically designed for ecological data and employs permutation tests to determine the most influential variables in a step-wise manner (120). The environmental variables selected by this algorithm were added to new models, and their significance was tested using Monte Carlo permutation tests in the vegan package. We selected models with the best predictive power based on their explained variability (*R*). The final RDA models were visualized using triplots in the vegan package.

Second, we calculated correlations among the cTPut, wTPut, and mite gene expression data sets independently for single-infected and mixed cultures using the Spearman correlation coefficient and bootstrap permutational *P* values in PAST. Only correlations with *P* < 0.05 were included in downstream analyses. We constructed correlation heatmaps using the Complex Heatmap package (121, 122) and clustered them using Ward distance or K-mean (in PAST) clustering for the interaction among symbionts and mite KEGG gene expression in different pathways. Fruchterman–Reingold network plots were prepared on PAST.

Our gamma diversity analysis was based on the identification of up- and down-regulated gene expression. FDR was calculated for single-infected and mixed samples for the cTPut, wTPut, and mite KEGG gene expression in the fuzzySim package (123, 124) and visualized as volcano plots. To construct the abundance heatmaps, we followed the same protocol as for the correlations heatmap.

## Data Availability

DNA and RNA sequences are deposited in GenBank (PRJNA493156, PRJNA690683, PRJNA656450, PRJNA990474, and PRJNA706095). Genomes are deposited in GenBank (JAUEML01, JAUEMM01, and JBBPFL01). The data are available as supplemental material and at https://zenodo.org/records/15172873 as Table S3_1 to S3_3.

## SUPPLEMENTAL MATERIAL

Supplemental figures msystems.01769-24-s0001.docx Figures S1-S16.

Supplemental tables msystems.01769-24-s0002.xlsx Tables S1-S15.

ASM does not own the copyrights to Supplemental Material that may be linked to, or accessed through, an article. The authors have granted ASM a non-exclusive, world-wide license to publish the Supplemental Material files. Please contact the corresponding author directly for reuse.
