# Supplementary material for: Cardinium disrupts Wolbachia–host dynamics in the domestic mite Tyrophagus putrescentiae: evidence from manipulative experiments
Source: mSystems. 2025 Apr 18;10(5):e01769-24. doi: 10.1128/msystems.01769-24 (PMC12090732; doi:10.1128/msystems.01769-24)
Supplement: Supplemental figures — Figures S1-S16. [file msystems.01769-24-s0001.docx]

**Supplementary figures S1–S16**

**Title:** *Cardinium* disrupts *Wolbachia*–host dynamics in the domestic mite *Tyrophagus putrescentiae* – evidence from manipulative experiments

**Authors:** J. Hubert, E. Glowska, S. E. Dowd, P. B. Klimov

**Journal:** mSystems

**Fig S1** Comparison of *Cardinium* (cTput) genomes in Proksee (95) using FASTANI (121). **A** – Comparison of our assembly (JAUEML01) of cTput to the Chinese cTput strain (JANAVR01), with average nucleotide identity (ANI) = 99.74. **B** – Comparison of our cTput assembly with that of *Cardinium* symbiont of *Sogatella furcifera* (cSFur) with ANI=91.96*.*


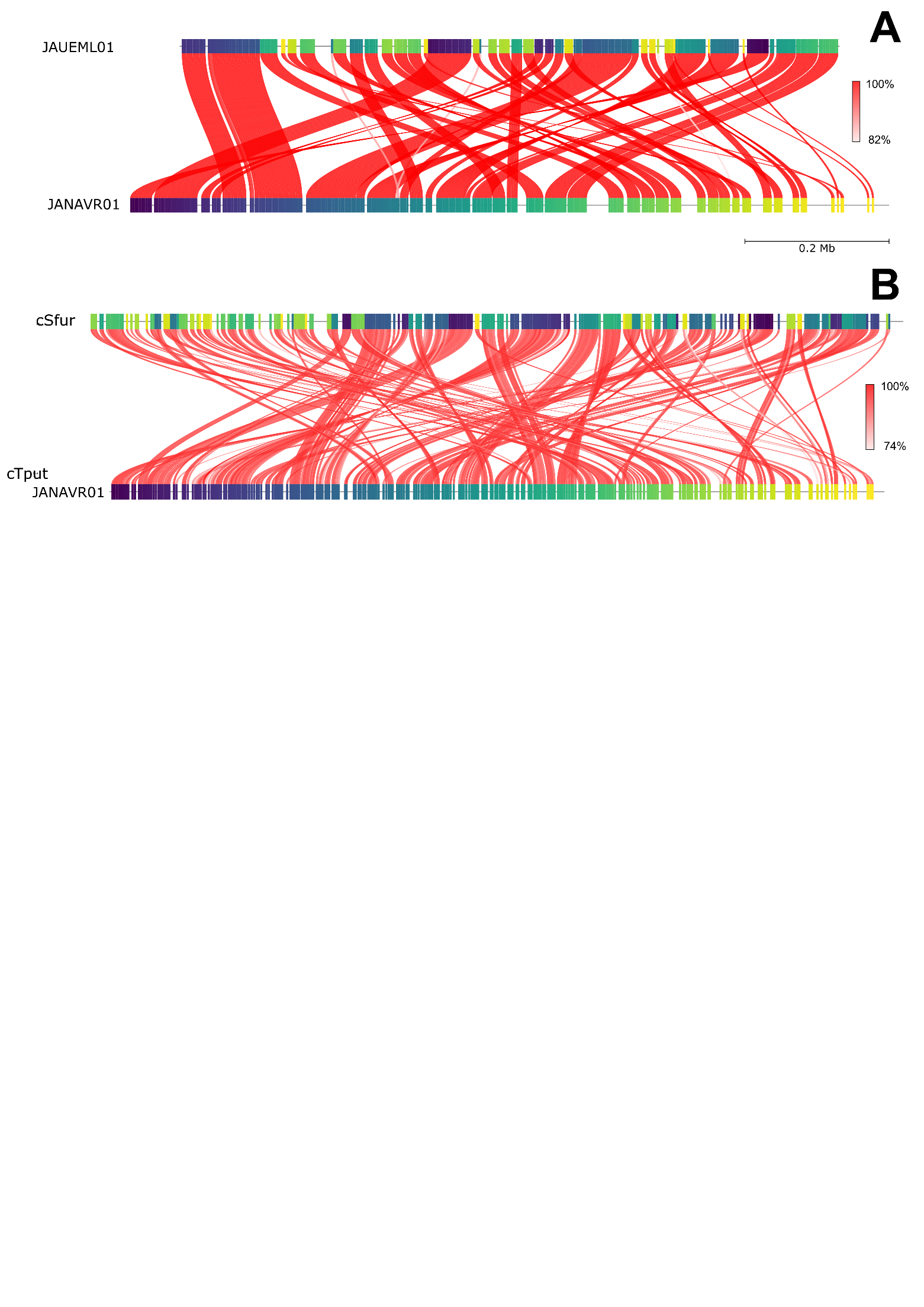


**Fig S2** Comparison of the *Cardinium* (cTput) genome (red point) to others *Cardinium* available in using the M1CR0B1AL1Z3R pipeline (49). GC content and open reading frame (ORF) comparisons are shown as violin and scatter plots.

**
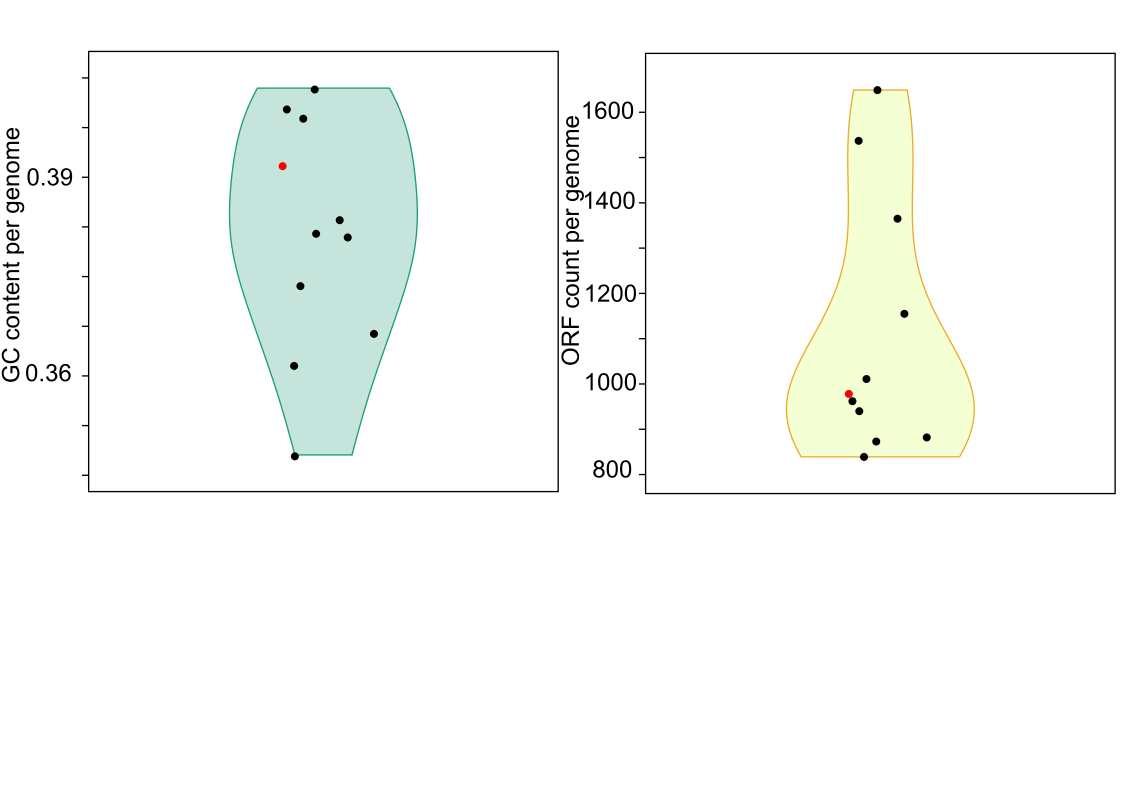
**

**Fig S3** Venn diagram comparing the KEGG assigned proteins of cTput from *Tyrophagus putrescentiae* CZ and Chinese strain (42)*,* *Sogatella furcifera* (15) and *Oedothorax gibbosus* (120).


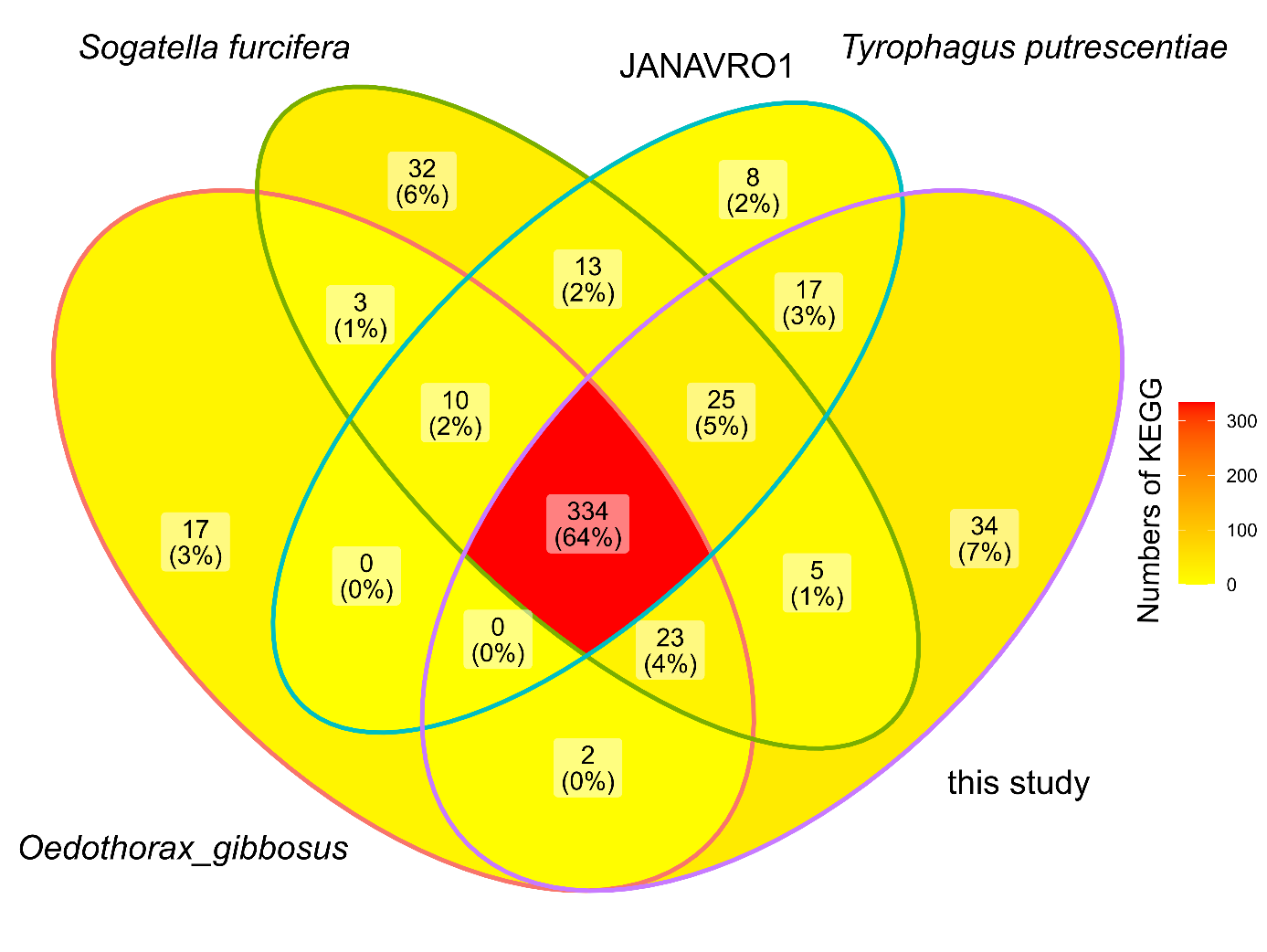


**Fig S4** Phylogenetic analyses of *Wolbachia* based on whole-genome similarity metrics using *Wolbachia* genomes available in GenBank; **A** – phylogram based on the M1CR0B1AL1Z3R pipeline. The dendrogram is organized according to *Wolbachia* host. **B** – FastANI analysis using average nucleotide identities (ANI); **Legend:** * -wTPut (*Wolbachia*) of *Tyrophagus putrescentiae*, ** GIJY0000000 annotation of wTPut from *T. putrescentiae*; *** identified *Wolbachia* species.

**A**


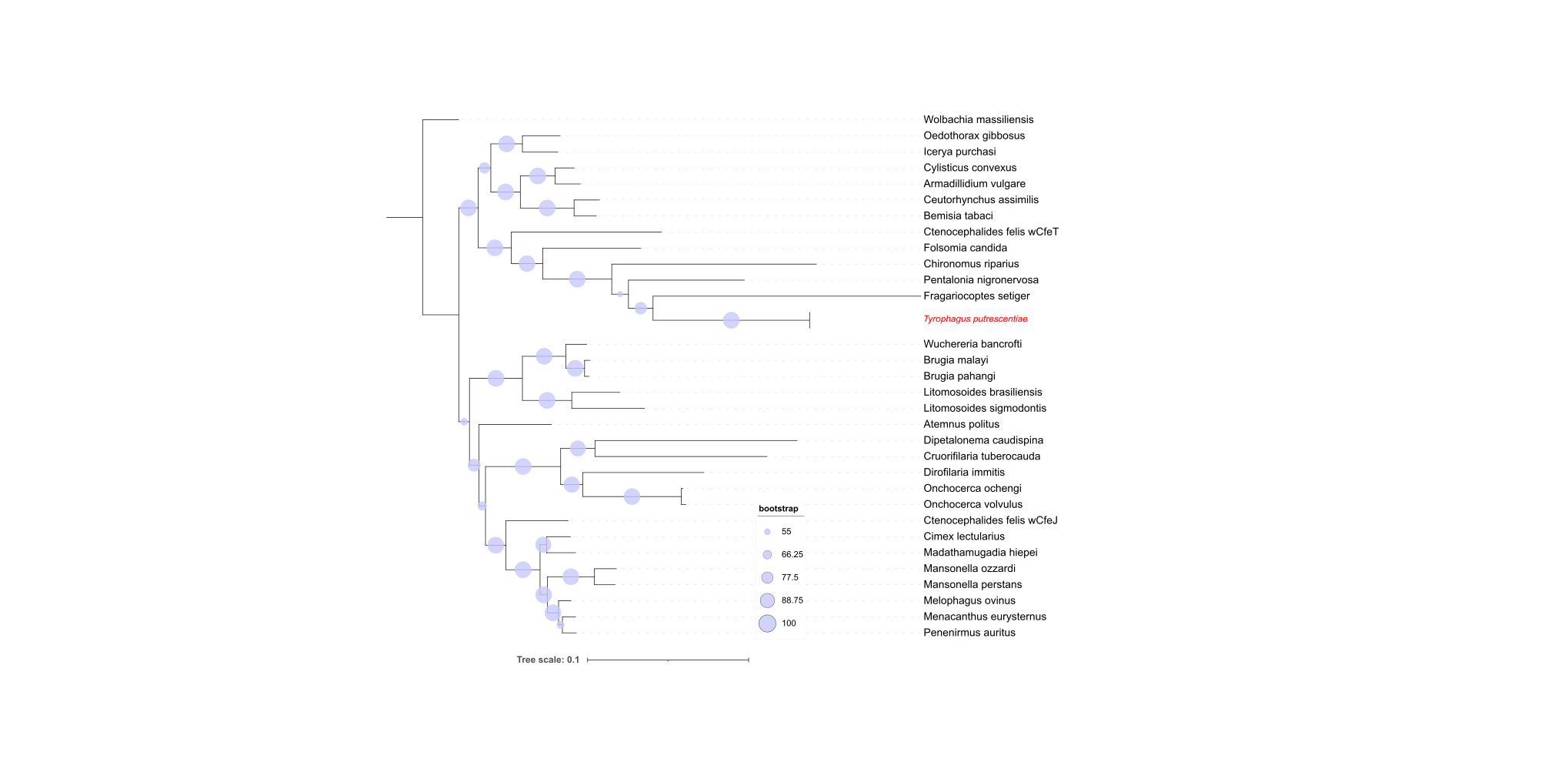


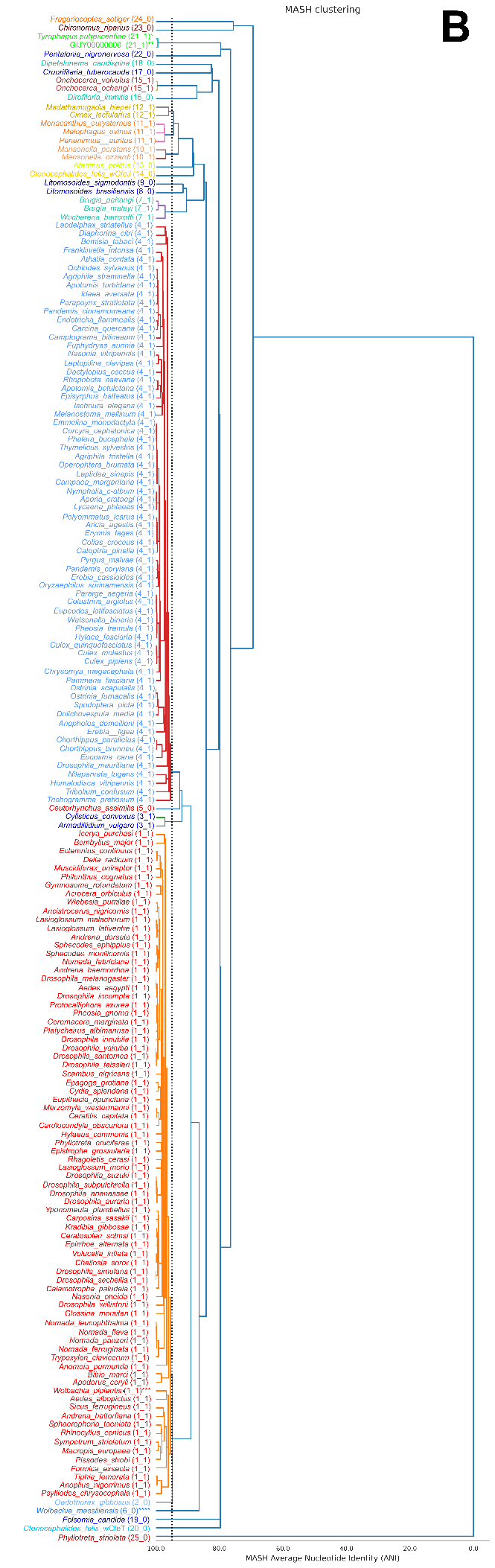


**Fig S5** Comparison of the *Wolbachia* (wTPu) genome of *Tyrophagus putrescentiae* (red dot) to others *Wolbachia* available in GenBank using the M1CR0B1AL1Z3R pipeline (49). GC content and open reading frame (ORF) comparisons are shown as violin and scatter plots. The blue point indicates the previous incomplete wTPu assembly.


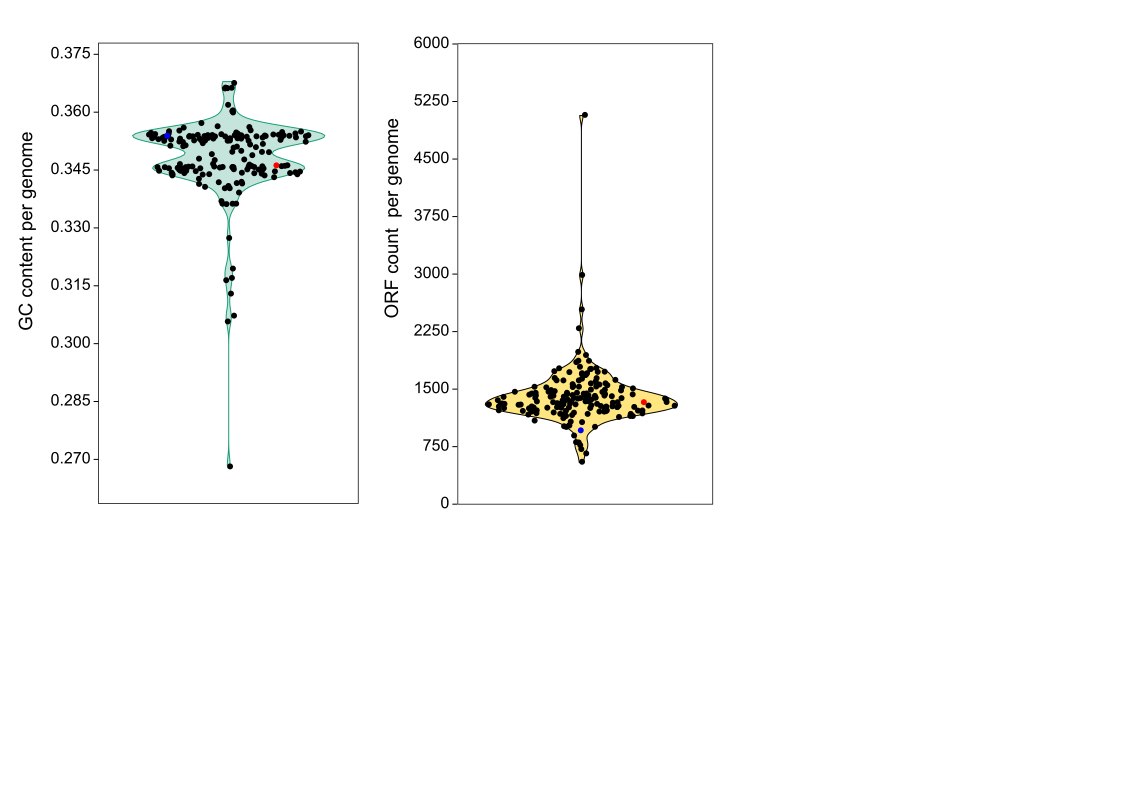


**Fig S6** Venn diagram comparing the KEGG assigned proteins of wTPu from the domestic mold mite *Tyrophagus putrescentiae* and the plant-feeding mite *Fragariocoptes setiger* (50) and *Pentalonia nigronervosa* (51); GIJY0100000 is a previous incomplete *Wolbachia* from *T. putrescentiae.*


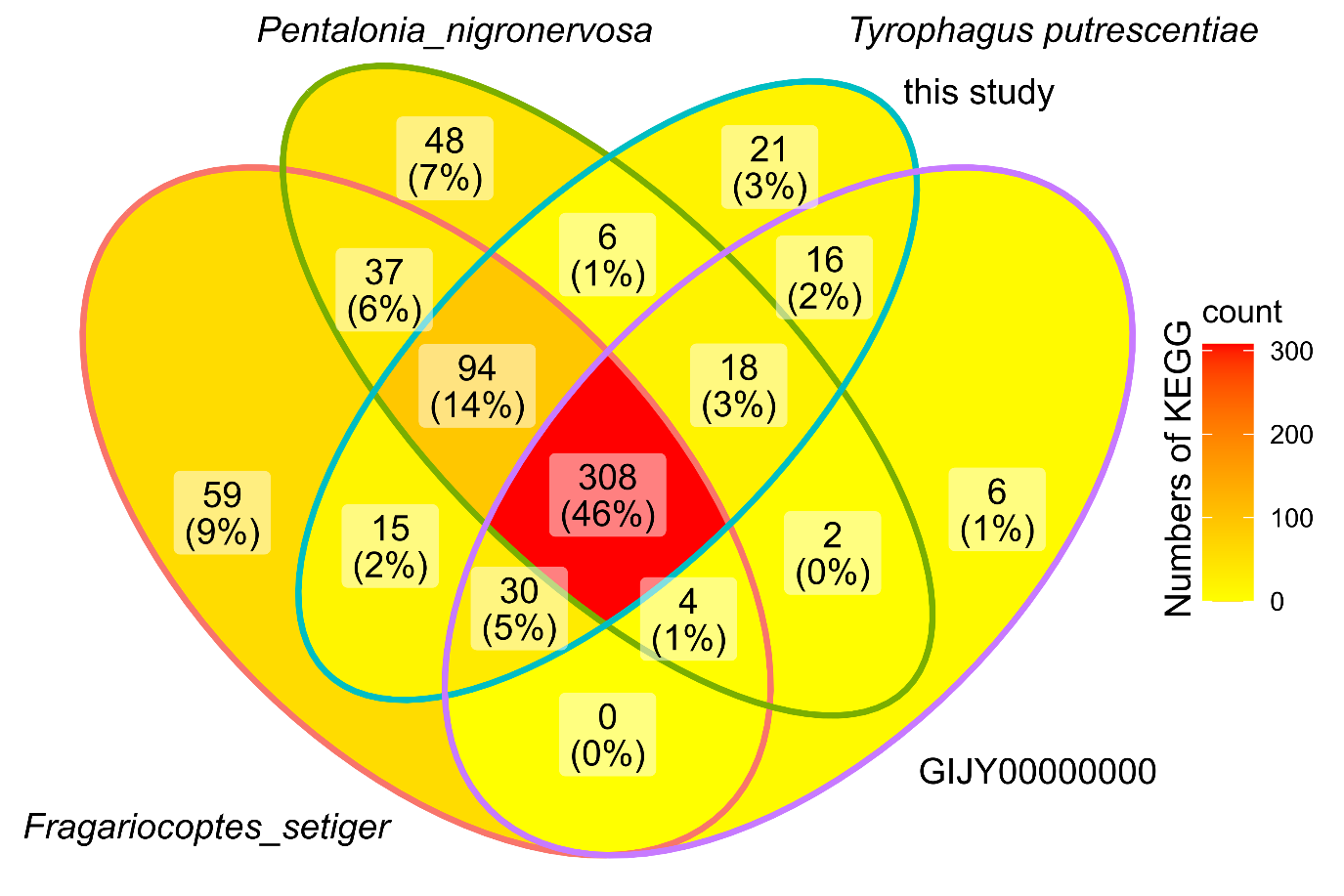


**Fig S7** Comparison of *Wolbachia* genomes in Proksee (95) using FASTANI (121). **A** – The comparison of new asseblage (JAUEMM01) of wTput to *Wolbachia* from mite *Fragariocoptes setiger* (JAHRAF01) with 77.16 average nucleotide identity (ANI). **B** – (JAUEMM01) of wTput to *Wolbachia* from *Folsomia candida* (NZ_CP015510) with 77.39 ANI*.*

**
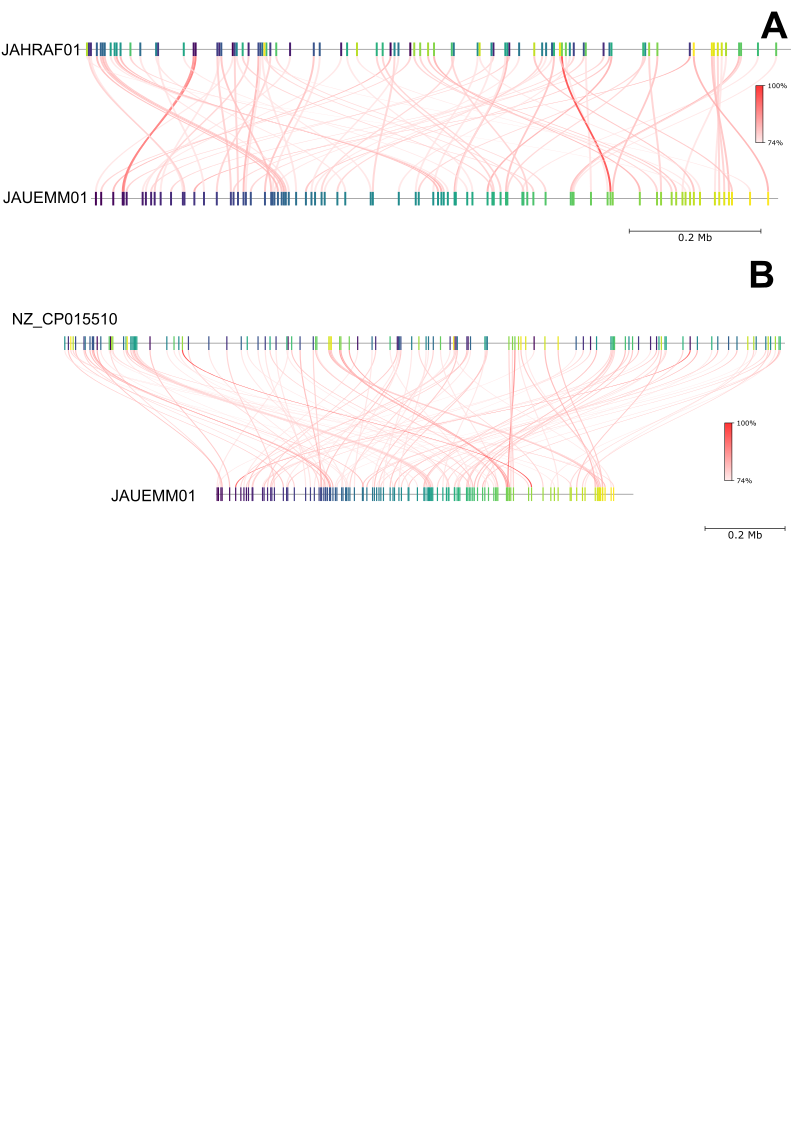
**

**
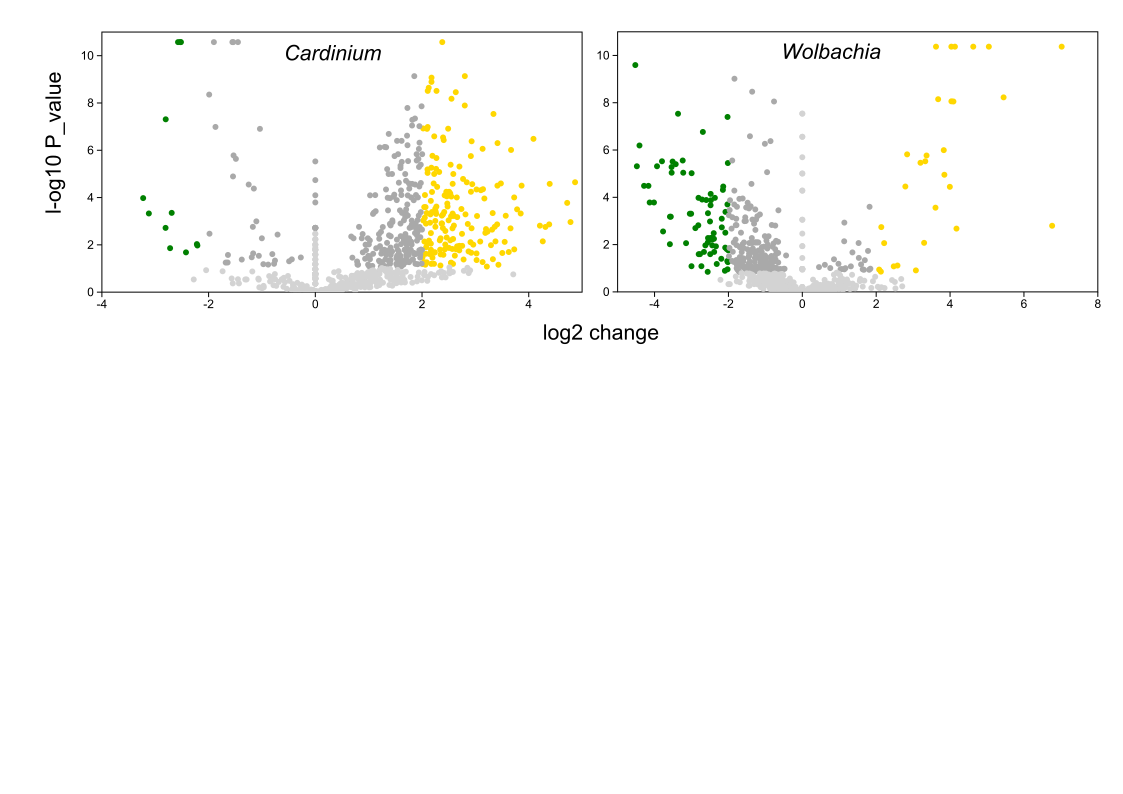
Fig S8** The comparison of genome expression of *Cardinium* and *Wolbachia* in single-infected and mixed cultures of mite host (*Tyrophagus putrescentiae*). The comparison is given in volcano plot, dark gray indicates genes with FDR P values >0.05; the rest of genes had P<0.05; the light grey points are those with log2fold changes <2; yellow colors indicate upregulated genes in double infected cultures, while green color downregulated.

**Fig S9** The correlation heatmap of selected bacterial proteins associated to ABC transporters, quorum sensing and bacterial secretion according to KEGG assign. The correlation heatmap for selected proteins is shown. The correlations between *Cardinium* and *Wolbachia* were calculated in mixed samples. The levels of Spearman correlations (P<0.05) are shown the most important predicted genes are marked by red color.

**Fig S10** The numbers of positive and negative correlations of *Wolbachia* predicted genes expression to *Cardinium* in mixed samples for selected proteins. The selected proteins were associated with ABC transporters, quorum sensing and bacterial secretion according to KEGG assign and total number of correlations (Spearman, P<0.05) are shown. The most important predicted genes are marked by red color.

**Fig S11** The numbers of positive and negative correlations of *Cardinium* predicted genes expression to *Wolbachia* in mixed samples for selected protein. The selected proteins were associated with ABC transporters, quorum sensing and bacterial secretion according to KEGG assign and total number of correlations (Spearman, P<0.05) are shown. The most important predicted genes are marked by red color.

**Fig S12** Analysis of similarities (ANOSIM) in gene expression of mite *Tyrophagus putrescentiae* host among single *Cardinium* (cTPut) or *Wolbachia* (wTPut) infected and mixed (double) cultures. The letters indicate difference (Bonferroni corrected p values) among the samples.

**
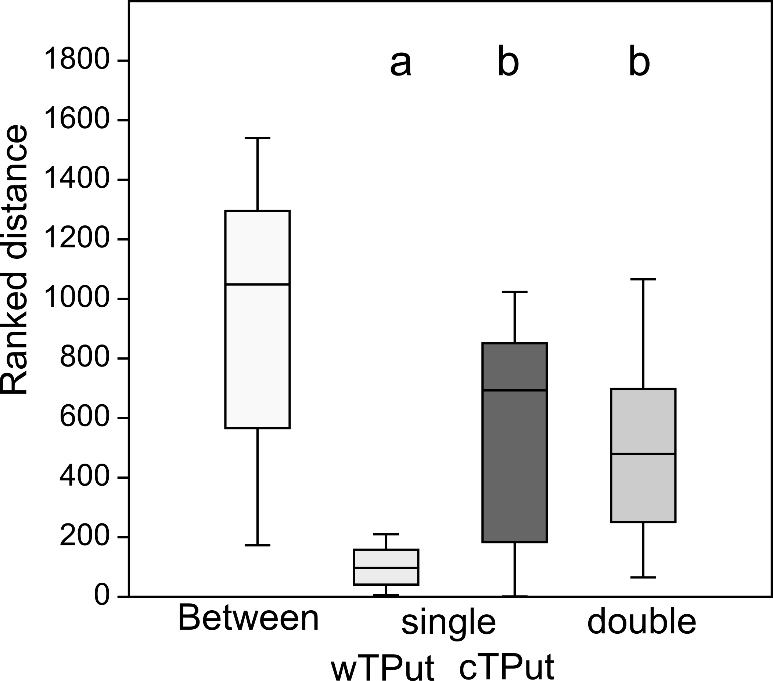
**

**Fig S13** A triplot of distance-based redundancy analysis (dbRDA) showing interaction among gene expression of *Tyrophagus putrescentiae* (KEGG genes) and its symbionts *Cardinium* and *Wolbachia* among different cultures. These mite cultures were infected either by a single bacterium, i.e *Cardinium* in 5L,5S; *Wolbachia* in 5P and 5N and mixed cultures (5LN, 5LP, 5SN, 5SP).


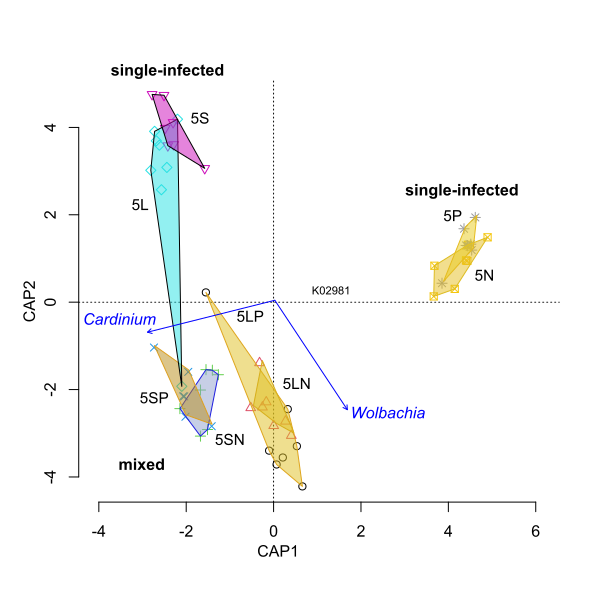


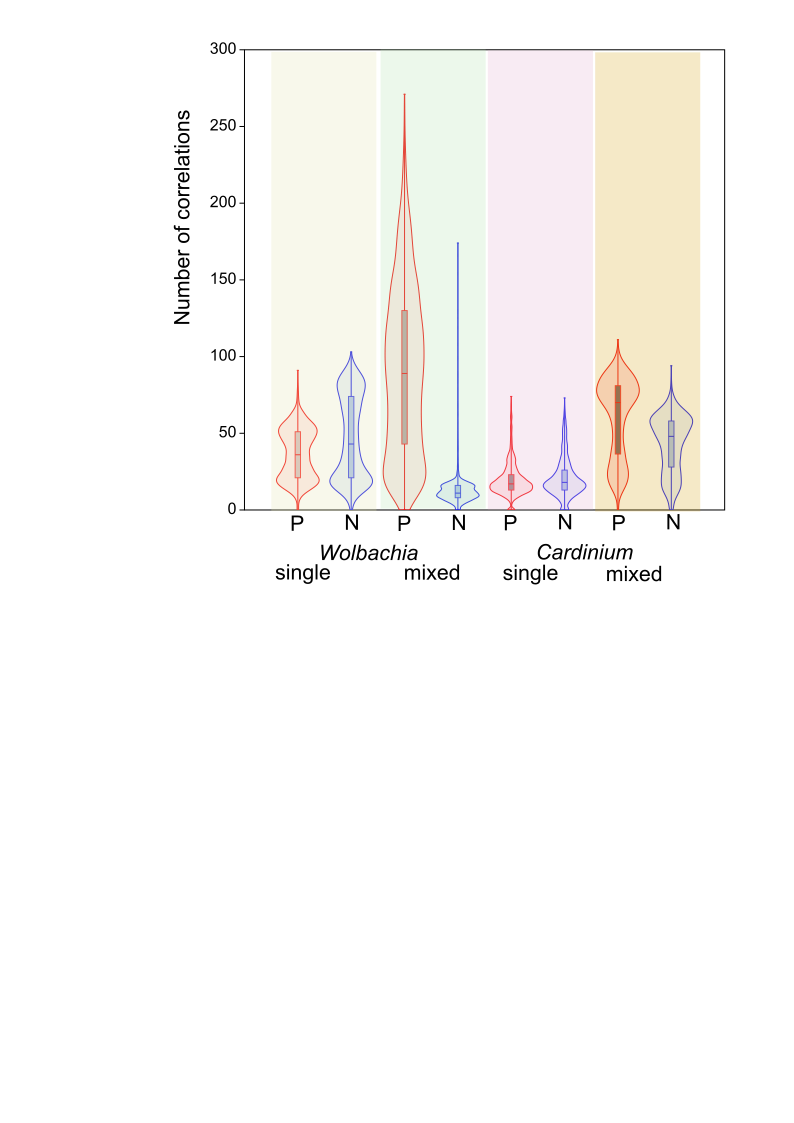
**Fig S14** Spearman correlations between gene expression of two bacterial symbionts and their host, *Tyrophagus putrescentiae*. Violin and box plots show the number of positive and negative correlations between mite KEGG genes vs *Cardinium* and *Wolbachia* in single- and double-infected cultures. Correlations were expressed per mite KEGG genes. All groups were significantly different from each other using Mann–Whitney test (P<0.002).

**Legend**: P – positive correlations; N -negative correlations


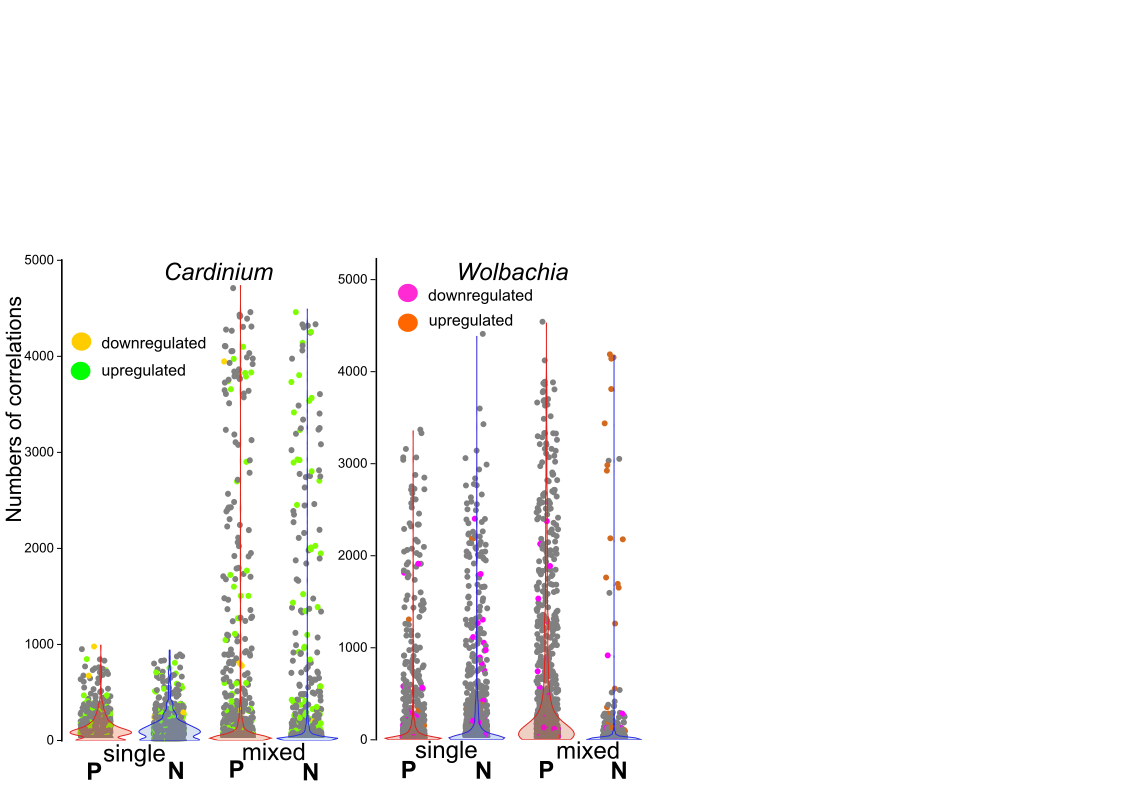
**Fig S15** Spearman correlations between gene expression of bacterial symbionts and their host, *Tyrophagus putrescentiae*. Violin and jitter plots show the number of positive and negative correlations between bacterial and mite KEGG genes in single- and double-infected cultures, expressed per bacterial gene. Up- and downregulated genes are indicated by different colors.

***

**Legend**: P – positive correlations; N – negative correlations

**Statistical analyses:** The significance of group differences was determined using sing test providing following results: *Cardinium* single versus double (i) positive r=478, P<0.001; (ii) negative r=527, P<0.001; *Wolbachia* single versus mixed (i) positive r=688, P<0.001, (ii) negative r=629, P<0.001.

**Fig S16** Identification of actin binding proteins (MDN5248151) from Wolbachia (wTPut). The tree was bootstrap to protein MVB089928 of the *Wolbachia* endosymbiont of *Fragariocoptes setiger.* The complete list of proteins in analyses is in Table S11.


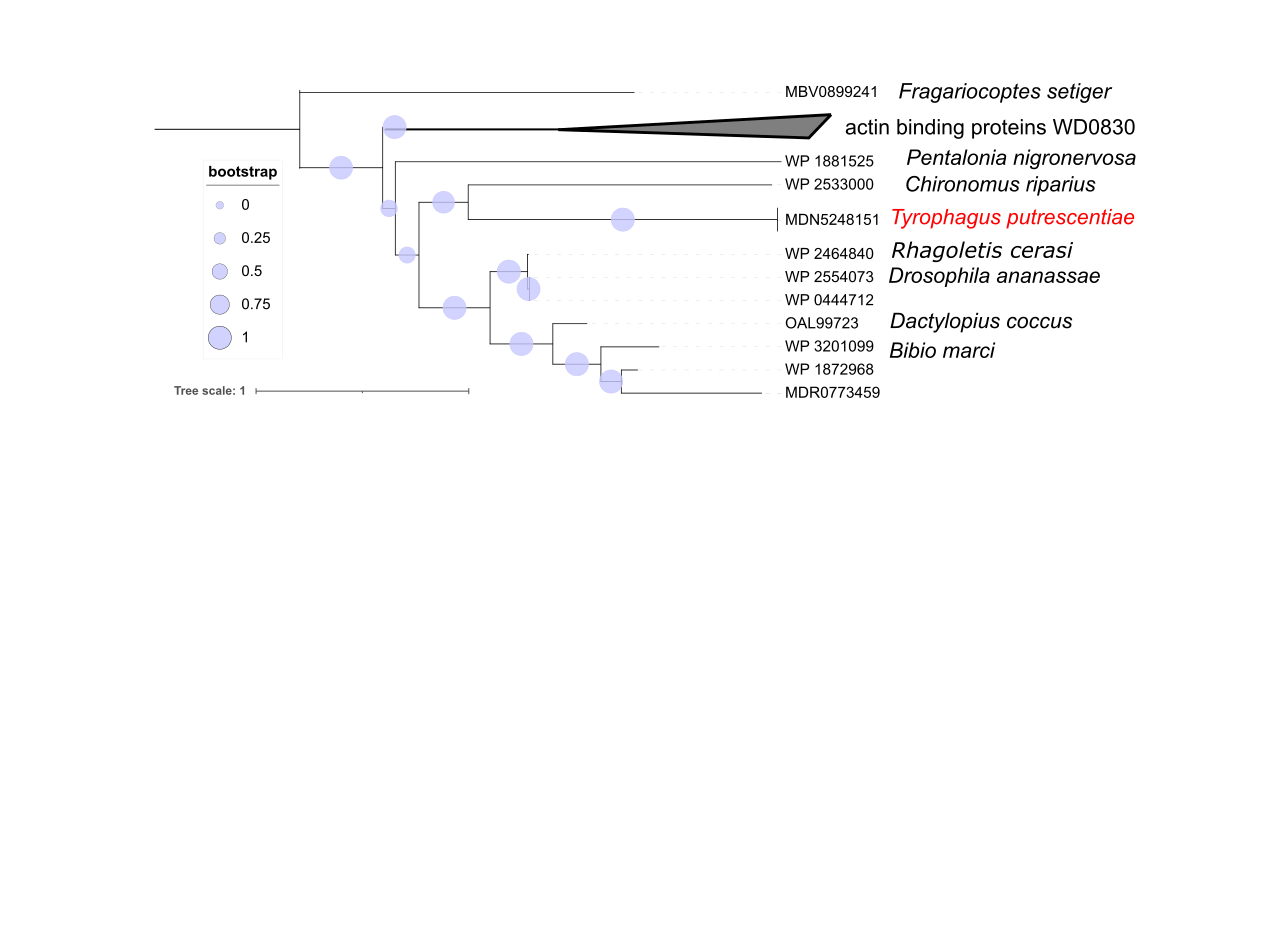


**Supplementary references**

15. Zeng Z, Fu Y, Guo D, Wu Y, Ajayi OE, Wu Q. 2018. Bacterial endosymbiont *Cardinium* cSfur genome sequence provides insights for understanding the symbiotic relationship in *Sogatella furcifera* host. BMC Genomics 19(1):688. <https://doi.org/10.1186/s12864-018-5078-y>

42. Xiong Q, Fung CS-H, Xiao X, Wan AT-Y, Wang M, Klimov P, Ren Y, Yang KY, Hubert J, Cui Y, Liu X, Tsui SK-W. 2023. Endogenous plasmids and chromosomal genome reduction in the *Cardinium* endosymbiont of *Dermatophagoides farinae*. mSphere 8(2):e0007423. <https://doi.org/10.1128/msphere.00074-23>

49. Avram O, Rapoport D, Portugez S, Pupko T. 2019. M1CR0B1AL1Z3R—a user-friendly web server for the analysis of large-scale microbial genomics data. Nucleic Acids Res 47(W1):W88–W92. <https://doi.org/10.1093/nar/gkz423>

50. Klimov PB, Chetverikov PE, Dodueva IE, Vishnyakov AE, Bolton SJ, Paponova SS, Lutova LA, Tolstikov AV. 2022. Symbiotic bacteria of the gall-inducing mite *Fragariocoptes setiger* (Eriophyoidea) and phylogenomic resolution of the eriophyoid position among Acari. Sci Rep 12(1):3811. <https://doi.org/10.1038/s41598-022-07535-3>

51. Mathers TC, Mugford ST, Hogenhout SA, Tripathi L. 2020. Genome sequence of the banana aphid, *Pentalonia nigronervosa* Coquerel (Hemiptera: Aphididae) and its symbionts. G3 (Bethesda). 10(12):4315–4321. <https://doi.org/10.1534/g3.120.401358>

95. Grant JR, Enns E, Marinier E, Mandal A, Herman EK, Chen C-y, Graham M, Van Domselaar G, Stothard P. 2023. Proksee: in-depth characterization and visualization of bacterial genomes. Nucleic Acids Res 51(W1):W484–W492. <https://doi.org/10.1093/nar/gkad326>

120. Halter T, Hendrickx F, Horn M, Manzano-Marin A. 2022. A novel widespread MITE element in the repeat-rich genome of the *Cardinium* endosymbiont of the spider *Oedothorax gibbosus*. Microbiol Spectr 10(6):e0262722. <https://doi.org/10.1128/spectrum.02627-22>

121. Jain C, Rodriguez-R LM, Phillippy AM, Konstantinidis KT, Aluru S. 2018. High throughput ANI analysis of 90K prokaryotic genomes reveals clear species boundaries. Nat Commun 9(1):5114. <https://doi.org/10.1038/s41467-018-07641-9>
